# Appraising research policy instrument mixes: a multicriteria mapping study in six European countries of diagnostic innovation to manage antimicrobial resistance

**DOI:** 10.1016/j.respol.2020.104140

**Published:** 2021-05

**Authors:** Josie Coburn, Frederique Bone, Michael M. Hopkins, Andy Stirling, Jorge Mestre-Ferrandiz, Stathis Arapostathis, Martin J. Llewelyn

**Affiliations:** a Science Policy Research Unit, University of Sussex Business School, University of Sussex, Falmer, Brighton, United Kingdom; b Visiting Fellow, Office of Health Economics, London, United Kingdom; c Department of History and Philosophy of Science, National and Kapodistrian University of Athens, Athens, Greece; d Brighton and Sussex Medical School, Falmer, Brighton, United Kingdom

**Keywords:** Multicriteria mapping, Policy instrument mixes, Antimicrobial resistance, Diagnostic innovation, Policy evaluation, Foresight appraisal

## Abstract

This article provides prospective appraisal of key policy instruments intended to stimulate innovation to combat antimicrobial resistance (AMR). AMR refers to the ability of microbes to evolve resistance to those treatments designed to kill them, and is associated with the overuse or misuse of medicines such as antibiotics. AMR is an emerging global challenge with major implications for healthcare and society as a whole. Diagnostic tests for infectious diseases can guide decision making when prescribing medicines, so reducing inappropriate drug use. In the context of growing international interest in policies to stimulate innovation in AMR diagnostics, this study uses multicriteria mapping (MCM) to appraise a range of policy instruments in order to understand their potential performance while also highlighting the uncertainties that stakeholders hold about such interventions in complex contexts. A contribution of the article is the demonstration of a novel method to analyse and visualise MCM data in order to reveal stakeholder inclinations towards particular options while exploring interviewees’ uncertainties about the effectiveness of each instrument's design or implementation. The article reports results from six European countries (Germany, Greece, Italy, the Netherlands, Spain and the UK). The findings reveal which policy instruments are deemed most likely to perform well, and why, across stakeholder groups and national settings, with areas of common ground and difference being identified. Importantly, the conclusions presented here differ from prominent policy discourse, with international implications for the design of mixes of policy instruments to combat AMR. Strategic and practical methodological implications also emerge for general appraisal of innovation policy instrument mixes.

## Highlights

• The following contributions are provided: A general process for conducting systematic prospective appraisal of policy options within the field of research policy, using multicriteria mapping (MCM).
• Novel methods for systematically analysing MCM data using pairwise comparison and merit orders which improve the utility of the MCM method.
• Quantitative findings (derived using MCM) on the appraisal of policy options for antimicrobial resistance (AMR) by 47 experts from six European countries, providing seven stakeholder perspectives, and identifying most and least favoured policy options.
• Qualitative findings (derived using MCM) addressing different modes of reasoning as to why contrasting policy options may be expected to perform well or poorly in different settings and from different perspectives.
• Qualitative findings (derived using MCM) underpinning a framework for policy design that may be useful for research policy around AMR.
• Quantitative findings (derived not using MCM) concerning those particular options that interviewees identified as most complementary to each other, supported by qualitative illustrations and qualifications.

## Graphical abstract

**Figure:**
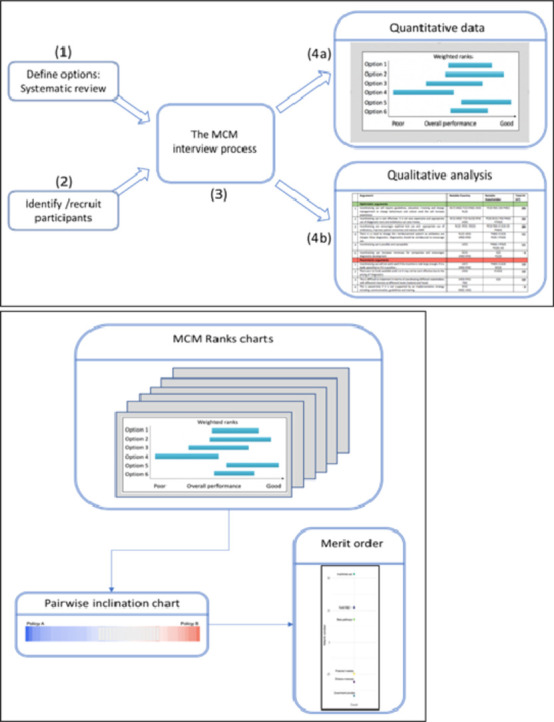

## 1 Introduction

This article provides prospective appraisal of key policy instruments intended to stimulate innovation to combat antimicrobial resistance (AMR), using a novel multicriteria analysis within a generalisable framework for the analysis of policy instrument mixes. In doing so, the article provides important insights for the study of innovation policy as well as offering a contribution to policy making in relation to an emerging global challenge for healthcare and society as a whole.

The establishment of Pasteur's Germ Theory in the late 19th century led to the development of a wide range of medicines targeting pathological microorganisms, leading to a great reduction in mortality rates from infectious disease globally (Porter, 1999; Kingston, 2000). However, resistance to antimicrobial drugs generally emerges following their widespread use, threatening therapeutic effectiveness (Sharma and Towse, 2011; Bell et al., 2014). AMR has recently been estimated to cause more than 23,000 deaths per annum in the USA and over 25,000 in the EU (CDDEP, 2015). Despite longstanding difficulties in estimating the current and future burden of AMR globally, such attempts are nonetheless associated with a scenario, widely disseminated by senior medical experts, of a global ‘antibiotic apocalypse’, which threatens the practice of modern medicine, whereby routine procedures such as chemotherapy and surgical operations would be too dangerous to undertake without anti-infective drugs (Smith and Coast, 2012, 2013; Davies et al., 2013; Wellcome Trust, 2019).^1^ The World Health Organisation (WHO), the European Union (EU), and many national governments have launched AMR action plans with aims including the reduction of antibiotic use in agriculture and human healthcare, as well as the ramping up of innovation for anti-infective drugs, vaccines and diagnostic tests (Federal Ministry of Health, 2008; DH and DEFRA, 2013; The White House, 2015; WHO, 2015; EHP Committee on AMR, 2016).

This article focuses specifically on the appraisal of policy instruments to support the development and use of diagnostic tests to manage AMR. Diagnostics are not generally the primary focus of AMR responses. For example, in European countries, R&D efforts have focused to a much greater extent on new therapeutics, including, but not limited to, new antibiotics (Kelly et al., 2016). Yet such therapeutics are expensive and slow to develop (Czaplewski et al., 2016). They also require public subsidies of hundreds of millions of dollars to stimulate commercial interest because many pharmaceutical firms have moved away from antibiotic R&D over recent years to focus on more profitable markets such as cancer (Sharma and Towse, 2011; Ferraro et al., 2017; Towse et al., 2017). Furthermore, resistance to new antimicrobial drugs tends to develop within a few years of introduction and correlates with levels of drug usage (ibid), suggesting that the development of new drugs alone will not provide a sustainable solution to the AMR challenge.

Diagnostic tests that can distinguish bacterial infection from other forms of illness and determine the presence of antibiotic resistance are important to provide public health surveillance and to guide the medical treatment of individuals, potentially avoiding unnecessary use or overuse of antibiotics (O'Neill, 2015). Such tests do not face the same regulatory hurdles as novel drugs (Phillips, Bebber and Issa, 2006) and so may reach the market more swiftly. However, a recent review of diagnostics for AMR concluded that innovation has been slow and the uptake of new tests has been limited (O'Neill, 2015). Many policy recommendations have been made to address this challenge (Spellberg et al., 2011; O'Neill, 2015; CDC and AdvaMedDx, 2016), but there is as yet little extant literature that provides evaluation of the effectiveness of such a wide range of policy options.

With so many countries facing the AMR challenge, this article aims to support the development of innovation policy to counter AMR by providing a novel method for policy appraisal based on multicriteria mapping (MCM), which is then applied to six European countries. The method gives diverse stakeholders in these six countries the opportunity to appraise policy options intended to stimulate diagnostic innovation in support of a national AMR strategy. The overarching question asks, by reference to a range of national settings and stakeholder perspectives, ***which policy instruments are expected to perform more or less favourably, under which conditions and why?*** Findings in this regard might support practical prioritisation of policy instruments in national AMR action plans.

Despite illuminating some crucial differences across both settings and perspectives, this study also identifies some notable common ground in terms of the outcomes of the appraisal, particularly across interviewees from the larger European countries. This picture of convergent stakeholder inclinations towards particular policy instruments – including their use in combination – is all the more compelling for arising from a method that attends so carefully to revealing differences in perspectives.

In this article we use the term ‘perspective’ to refer to “a grouping of viewpoints that may be seen on the basis of MCM analysis to display certain features in common” (Coburn et al., 2019). Individual viewpoints can be grouped into perspectives in different ways, e.g. by their country setting or by their stakeholder group.

Applying MCM in a new field and manner, the article also tests a novel general method for appraising prospective policy instrument mixes. A crucial feature of this approach is the systematic exploration of uncertainties across diverse settings and stakeholder perspectives. Respecting key principles both of quantitative and qualitative rigour, this process provides a picture of comparative performance that is both systematic and reproducible in its scope, as well as more nuanced in its attention to ever-present subjectivity and conditionality (Goertz and Mahoney, 1996; Mingers, 1997; Shah and Corley, 2006; Porta and Keating, 2008; Delyser and Sui, 2014). Section 2 sets out the associated conceptual framework for prospective appraisal of innovation policy options and discusses practicalities for its implementation. A series of sub-questions are identified, which stem from the framing of the overarching research question above in the innovation policy literature. Sections 3 and 4 introduce the research design and methods used. Section 5 reports the findings of the empirical research, while Section 6 provides the discussion of these results both in the context of wider efforts to address AMR and the literature on the evaluation of innovation policy more generally. Conclusions are drawn on the implications of the findings, noting limitations of this study and opportunities for further research.

## 2 Appraising innovation policy instruments

This article's aim is to identify those changes in policy that might best enable the most effective forms of innovation in AMR diagnostics across contrasting national settings and stakeholder perspectives. To meet this aim, it is necessary to address certain challenges in conceptualising and analysing innovation policy. Here, a range of approaches across a wide literature (OECD, 2010), suggests a short but usefully broad understanding of ‘innovation policy’ (in any given area), as “*all policies that have an impact on innovation*” (in that area) (Fagerberg, 2016). This includes policy impacts that may be indirect as well as direct (Dutrenit et al., 2013) and unintended as well as deliberate (Fagerberg and Mowery, 2009), and it requires going “*beyond the sectoral approach*” (OECD, 2005) to focus on the most relevant “*policy mix*” (Rogge and Reichardt, 2013) for promoting (in this case) effective innovation in AMR diagnostics. So, even under a particular viewpoint or in a very specific setting like this, the task is not simply about identifying a single ‘best policy’ (Kern and Howlett, 2009), but exploring an array of alternatives and their various possible conditions, interactions and contextual implications (Rogge and Reichardt, 2016).

A further challenge rests on the levels of diversity, uncertainty, contestation and historic path-dependency that typically affect innovation policy (Smits, Kuhlmann and Shapira, 2010) – especially in a field as complex as AMR diagnostics (Nesta, 2018; WHO, 2018; DHSC and DEFRA, 2019). This complexity extends even to notionally singular ‘policies’ which should (for the sake of rigour and accuracy) be recognised actually to comprise a number of more specific ‘instruments’ (Cunningham et al., 2013). Moreover, performance of each of these will typically be context-dependent, as instruments interact in complex ways (Rogge and Reichardt, 2016), resulting in complementarities and conflicts (Flanagan and Uyarra, 2016). Some instruments may depend on other instruments or particular local resources in order to be effective (Gunningham and Sinclair, 1999; Cunningham et al., 2013). Solutions in one country or system cannot therefore be expected to ‘travel well’ to others because their effectiveness is dependent on so many contextual interactions (Nelson, 2008; Mazzucato, 2018).

Similarly no ‘one-size-fits-all’ instrument mix is to be expected, even when looking at the same problem in different contexts (Borrás and Edquist, 2013). This may be because instruments are selected and their design is shaped locally by policy makers in response to the needs of powerful lobbies, political ideologies and macroeconomic conditions (Nelson and Winter, 1982; Borrás and Edquist, 2013; Hopkins et al., 2019). Moreover, different mixes of instruments are warranted over time, following the maturity of the market and changing bottlenecks, such as missing capabilities or connectivity, and these require attention at different times and perhaps involving different actors (Neij, 1999; Cunningham et al., 2013). The granularity of any enquiry seeking to identify the best array of interventions for promoting effective innovation in AMR diagnostics is thus most robustly constituted not by ‘innovation policies’ in general, but more precisely at the level of what might be called the relevant ‘policy instrument mix’ (Borrás and Edquist, 2013; Kivimaa and Kern, 2016). It is therefore at the level of specific instruments, rather than more general policies, that this article begins.

Even so, however, no single instrument – let alone any mix – can be so definitively specified, nor any context so carefully defined, that these are not in turn also seriously complicated by many intrinsic and unavoidable forms of variability, uncertainty and complex dynamics over time (Edmondson et al., 2018). Furthermore, efforts to provide rigour and precision in the characterisation of instruments, will nonetheless likely be perceived with ambiguity across diverse perspectives (Rogge and Reichardt, 2016). As a result, radically divergent stakeholder understandings (and thus appraisals) can be expected (Raven et al., 2017), with each apparently equally salient, valid and well-informed (Stirling, 2010).

Methods of appraisal should address in a systematic and rigorous way the challenge of divergent stakeholder views (Stirling, 2008b). This may involve resisting well-recognised pressures for justification in policy appraisal (Genus and Stirling, 2017), where political pressures to deliver single ‘simple answers’ and ‘elevator messages’ may force suppression of deep uncertainties and ambiguities. ‘Risks’ for instance, are misleadingly aggregated using probabilistic techniques, potentially providing erroneous impressions of confidence (Funtowicz and Ravetz, 1990; Wynne, 1992). In such ways, the ‘closing down’ of policy discussion may result from misleading assertions of a particular view as if it were somehow distinctively valid or robust (Stirling, 2008b).

It is unfortunate in the face of such challenges, that both retrospective evaluation and prospective appraisal of instrument mixes presently remain at an early stage of development (Cunningham et al., 2013). Indeed, it has recently been pointed out that system-oriented innovation policy more generally, remains an under-researched topic (Borrás and Laatsit, 2019). Yet, the importance of tailored and holistic thinking for instrument mixes is – for all the above reasons – increasingly compelling. For example, the OECD (2016b) reports that there is a global trend towards countries moving away from generic ‘policy mixes’ towards more tightly-focused sector- and technology-specific interventions, integrating both supply and demand sides. Here, there is an especially intense need for methodological innovation in order to address urgent needs for robust approaches to support developing instrument mixes for innovation policy (Borrás and Edquist, 2013; Cunningham et al., 2013). It is against this background that this article seeks to contribute to the prospective appraisal of policy mixes for AMR diagnostic innovation.

To this end, the above discussion suggests a number of methodological design criteria to make the proposed appraisal as rigorous and robust as possible. Taking each of the above points in turn, these criteria might be summarised as follows.

First, the methods used should be ***holistic*** in scope, capable of addressing a diverse array of contexts without undue privilege to any particular setting (e.g. high- or middle-income countries, ‘free market’ or more regulated economies). Second, the methods should be able to address a ***diversity*** of options, without unduly favouring particular kinds of intervention (e.g. public or private, supply- or demand-side, or technology- or organisationally based innovations).

Third, rather than being hardwired to identify a notionally single ‘best’ prescription, appraisal should be capable of addressing interactions, complementarities and tensions across ***portfolios*** of possible options (i.e. leaving open the possibility for finding mixes, not single interventions). Fourth, the approach should engage with salient ***conditionalities*** in respect of particular features of options, contexts or the unfolding of time (e.g. interrogation at the granularity of particular instruments rather than general policies).

Fifth, policy appraisal should avoid pretensions at deriving a supposedly uniquely ‘objectively valid’ picture – and instead give balanced attention to a ***plurality*** of relevant specialist understandings and perspectives (e.g. fairly engaging disparate stakeholder interests). Sixth, with respect to each such perspective, the method should be capable of addressing ***uncertainties*** (e.g. exploring the full range of possibilities for how innovations or their contexts may unfold over time, without artificial probabilistic aggregations).

More generally, these methodological design criteria suggest a need to appreciate limits to probabilistic techniques, which is also relevant in interpreting policy-relevant results (Collingridge, 1982), as will be seen later. In any complex policy appraisal, ambiguities arise concerning the dimensionalities, definitions and partitioning of categories of interest (like relevant populations, salient parameters, causal mechanisms or frequency distributions) (Stirling, 2010). Even when proposing categories of interest, eliciting whether the effects of an intervention apply to all categories and conditionalities does not lie in ‘statistical representativeness’ (Rothman et al., 2013). This means not only that this conventional aim in much quantitative research is difficult to achieve in practice, but also that statistical representativeness would not fully enable the exploration of conditionalities nor satisfy democratic representativeness (O'Neill, 2001).

Taking these considerations together, this article not only seeks directly to address the concrete – urgent and imperative – global policy challenge of supporting innovation in diagnostic testing to mitigate AMR, but also to address an additional methodological aim. In resolving the above criteria, it sets out to build on past practice in order to pilot a particular novel framework for prospective appraisal of innovation policy options, which attempts to address all the aforementioned demanding challenges. To the extent the above six criteria can be respected, the resulting framework should be of relevance in principle to decision making processes across a wide array of innovation policy challenges.

## 3 Process for the prospective appraisal of innovation policy instrument mixes

### 3.1 Design of method

For reasons well-articulated by Borrás and Edquist (2013), this study tests an implementation of the three stage process advocated for prospective appraisal of instrument mixes in innovation policy design. These stages comprise: (1) selection of instruments from a wide range of possible candidates; (2) customisation of these instruments to fit with particular aspects of relevant contexts; (3) design of specific resulting mixes of instruments. While Borrás and Edquist (2013) provide an overarching framework for innovation policy design there has been little attention to the methodology for undertaking systematic prospective appraisal of innovation policy instruments (Fagerberg, 2016).

It is recognised, including by Borrás and Edquist (2013), that such an approach should be based within a wider framework for ‘strategic foresight’ of innovation policy (Velamoor, 2000). Here, possible interventions should be appraised by a diverse range of stakeholders with salient expertise in relevant settings (Giaoutzi and Sapio, 2013). It is in this context, that the six specific criteria mentioned above are emphasised as central to designing the method of appraisal for instrument mixes, such as for diagnostic innovation to manage AMR.

An array of potentially relevant approaches have been developed explicitly in broad fields for policy appraisal (Roe, 1994; Nagel, 2002), decision analysis (Edwards, Miles and Winterfeldt, 2007), strategic intelligence (Kuhlmann, 2002), research evaluation (Fahrenkrog et al., 2002), innovation management (Shane, 2008) and technology assessment (Rip, Misa and Schot, 1995; Grin et al., 1997; Sclove, 2010). Despite many differences of detail – and a measure of ‘boundary work’ between disciplines and policy sectors (Yoshizawa, 2007) – a wide subset of these offer relevant frameworks for present purposes that: (a) characterise diverse possible options towards some focal policy goal; (b) distinguish the issues, values or settings most relevant to appraising these options; (c) explore the pros and cons of each option across relevant circumstances; (d) do all this under each of a number of divergent evaluative perspectives by involving a wide range of participants; (e) in order to yield a general merit ordering across all included options that takes these aspects into account (Ely et al., 2014).

With respect to the policy challenges highlighted in the last section, however, the resulting specific methods differ in their various abilities to: (f) maximise the agency of participants in framing their own appraisals and how they are interpreted and represented; (g) giving balanced consideration to qualitative as well as quantitative information; (h) deliberately elicit relevant uncertainties under each included perspective; (i) clearly illuminate ambiguities that arise from comparing divergent perspectives; (j) explicitly focus on trade-offs between different evaluative criteria (or alternative ways of being rational about contending priorities); (k) examining the extent and ways in which different options might relate to each other in a mix; and (l) respecting needs for transparency and accountability to third parties concerning the particular reasons why appraisal results take the forms they do (Ely et al., 2014).

Among the relatively few approaches that maximise these more specific qualities for policy appraisal, the well-established multicriteria mapping (MCM) method is prominent and favourably reviewed (POST, 2001; Yearley, 2001; Anon, 2004; Dodgson et al., 2009) across scores of policy appraisals undertaken in many sectors and countries (Stirling and Mayer, 2001; Burgess, 2004, Burgess, 2007; Stirling et al., 2007, 2008a; Brooks et al., 2009; Eames and McDowall, 2010; Hansen, 2010; Morgan Jones, 2010; Bellamy et al., 2013; Raven et al., 2017). MCM shares with other decision analysis methods the core elements of rigour listed under features (a) to (e) above, whilst also adopting an approach as open and unconstrained as possible in addressing the additional qualities (f) to (l) (Coburn and Stirling, 2016). Indeed, MCM has been specifically designed to enable both the ‘*broadening out*’ of the scope of what is taken into account (as *inputs* to policy appraisal) (Stirling, 1999); as well as the ‘*opening up*’ of the picture that is conveyed to wider political discussion (as *outputs* from appraisal) (Stirling, 2005). This quality of ‘opening up’ applies not just to engagements with ‘decision-makers’, but also with other actors in the wider political debates in which decision making is set (Stirling, 2008b).

Developed further in a number of distinctive ways for the purpose of the present project, the MCM process described in the following section enables the addressing of each of the six criteria discussed in the previous section for prospective appraisal of policy instrument mixes.

First, MCM is strongly *holistic in scope*, in that appraisal criteria are freely developed by interviewees such as to address an unfettered array of policy considerations as they see them – without being driven or blinkered by the analyst's own bias. Second, MCM adopts a balanced approach to a *diversity of options*, in that an initial set of core policy options are chosen by researchers for appraisal by all interviewees, such as to reflect a full range of axes of contrast in wider policy debates – with each interviewee free to add whatever additional options or changes of definition that they wish.

Third, MCM explicitly permits a *focus on portfolios* of options rather than just individual options (Stirling, 1997; Yoshizawa et al., 2011) – a feature specifically applied in the present study. Fourth, the emphasis of MCM on attention to qualitative nuances in appraisal (as understood by different specialist perspectives), gives it a relatively high sensitivity to *detailed conditionalities*. Fifth, the priority placed in MCM to ‘*putting the interviewee in the driving seat*’ gives general confidence of its ability to engage in an unbiased way across a *plurality of perspectives.* Finally (in relation to the sixth criterion in the last section), MCM focuses throughout, not just on eliciting under each perspective a supposedly singular scalar representation of relative merit, but also on *illuminating deep uncertainties.*

The above points suggest that MCM is a method well placed to address the demanding challenges of prospective policy appraisal as identified in the literature on policy instrument mixes. Indeed, to the extent that similar quality criteria apply, it might also be judged suitable for similar reasons, for retrospective policy evaluation. It is in testing the utility of a newly-developed version of this method in relation to these criteria, that a contribution to the practice of evaluation of innovation policy can be made at the same time as providing a rich empirical picture of relevant particularities across six European countries to inform the design of policy instruments to support AMR diagnostic innovation.

Having identified a process and a method, the remaining part of this section focuses on some practical considerations to be addressed in the implementation of the method.

### 3.2 Selection of core instruments for appraisal

A preliminary question to address is which options should be included for appraisal by stakeholders. An immediate challenge is that an “ocean” of innovation policy instruments exists (Borrás and Edquist, 2013) which can be classified in a number of ways (Steinmueller, 2010; Borrás and Edquist, 2013; Rogge and Reichardt, 2016). The first step is therefore to focus on an appropriate subset. There are various frameworks that can inform such a selection.

A commonly used three-fold categorisation distinguishes between (i) economic and financial instruments (ii) regulatory instruments and (iii) information (or soft) instruments – with these three being termed ‘carrots, sticks and sermons’ (Borrás and Edquist, 2013; Rogge and Reichardt, 2016).

Instruments can also be classified according to the functions of the innovation system, or the governance niche, that they target (Hekkert et al., 2007; Hopkins et al., 2019). In combination with the above, this approach rapidly generates dozens of potential categories of instrument (Borrás and Edquist, 2013). Moreover, it is immediately obvious that different facets of instrument design can further distinguish instruments that at first seem to fit within the same category; additionally, instruments may also perform more than one role (Hopkins et al., 2019), and so address more than one ‘box’ in a given matrix of instrument types.

Yet more classificatory considerations include: whether an instrument focuses on the supply-side or demand-side of a market (Borrás and Edquist, 2013; Rogge and Reichardt, 2016), often simplified as ‘technology push’ or ‘technology pull’ respectively; whether it addresses a (downstream) near market or upstream activity; and whether the actors driving the direction of innovation are public or private (Mazzucato, 2013).

As in any policy analysis, theoretical sophistication must at some point compromise with the practical necessity to engage with diverse stakeholders, who have limited analytical time to study the different options and finite bandwidth to address the cognitive demands of decision analysis methods. This inevitably imposes constraints on the range of options that can be considered. Yet to address the quality criteria set out above, it is necessary to minimise artificial limits on the kinds of instruments included in appraisal. This dilemma can be reconciled by studying options at the level of ‘families’ of instruments – where the term family implies that specific included instruments share features such as their target and mechanism, while still being potentially variable in configurations of other characteristics. The identification of important ‘family’ characteristics allows multiple instruments to be appraised as a single policy option, so that comparisons can be directed at major shared features in modes of action with respect to an innovation system. Detailed appraisal by participants at the level of specific features of particular instruments can then be focused at a particular point in appraisal under a specific perspective.

A further consideration in instrument definition, is not to select options that are merely theoretically possible, but to take seriously the history, context and politics (path dependency, agency and power) that have played a role in making certain options prominent in ongoing debates. For example, on the topic of policy options for managing AMR, a considerable literature exists (summarised in the supplementary materials: Annex A) in which powerful actors strongly advocate particular policy approaches. Such established options need to be acknowledged, but are most rigorously appraised alongside alternatives. This study provides stakeholders with a diverse portfolio of policy options for appraisal, assembled following a comprehensive review of relevant policy literature (discussed further in Section 4.1).

### 3.3 Customisation of instruments to fit context

To address this task, stakeholders can be engaged in order to identify both the general options and their salient variations, as favoured (or not) in relation to contrasting appraisal criteria. Here, contrasting participant appraisals are ‘plural and conditional’ as individuals will be able to identify conditions under which an option can perform well or poorly (Stirling, 2006). The detailed positive and negative considerations that arise more generally can – despite crucial differences across contexts – offer insights for more localised design of policy options.

In this international comparative study, the focus is on identifying policy options for national strategies to stimulate the diagnostic innovation needed to tackle AMR. Given the diverse national settings and perspectives that any such analysis inevitably spans, a key task lies in addressing the general quality criteria set out above, asking which features of policy options are variously favoured and disfavoured by different stakeholder groups under different conditions? It is in addressing this challenge, that the general point made above also applies, in that the qualitative reasons expressed as to why appraisals take the position they do on any given detail, are as important as the quantitative representations of relative orderings across options.

In order for this demanding criterion to be respected, salient features of options need to be defined in a manner that provides consistency at the same time as allowing stakeholders to appraise under whatever issues they consider to be most salient (with as little influence from the analyst as possible). What might count here as a ‘salient feature’ cannot comprehensively be anticipated *ex ante*, by the analyst, but will arise as an empirical result of the analysis itself. Yet this raises a further challenge, in that the greater the detail provided in advance by the analyst, the less generalizable appraisal can be across contexts and the higher the risk that interviewees will focus on minutia rather than broad principles. Stakeholders are therefore provided in MCM with a definition for each option that is only as detailed as can be retained in mind, for the purpose of a one- or two-hour intensive appraisal process (interview materials are provided in the supplementary materials: Annex B). Features of this description that come to the fore in different ways – like the envisaged mechanism, its intended target activities, the site of these in the innovation system and the roles the instrument requires for the state or industry to play – can all then be noted insofar as they become relevant across different appraisals. Moreover, just as a stakeholder can define their own additional option, where they believe something of relevance has been missed, so too can they add whatever they might consider to be meaningful variations in definitions for those options that have been included.

It is in these ways, that the definitions of options for appraisal in the MCM framework adopted in this study can address the demanding quality criteria described in Section 2 above. And it is on this basis, that the subsequent process can address the crucial additional challenges concerning variabilities across contexts, uncertainties about the future, ambiguities across perspectives and interactions between different instruments in a mix. In all these respects, the ability of MCM to compare appraisals under contrasting specialist perspectives, offers a greater basis for confidence in the resulting findings, than would be the case if the appraisal were (as is often otherwise the case) solely based on the understandings and interests of the analysts alone.

### 3.4 Implications for the ‘instrument mix’

As was discussed in detail in Section 2 above, a crucial factor in appraisal is the attention paid not only to the conditionalities associated with specific instruments across contexts and perspectives, but also to the ways in which they may be expected to interact in a ‘policy mix’. It is partly for these reasons that the term ‘instrument mix’ is increasingly preferred in the literature to the more coarse-grain idea of a ‘policy mix’. But – as also discussed in relation to the quality criteria above – this presents an additional demanding challenge in appraisal. If attempts are made to address this in any encyclopaedic way, the sheer numbers of permutations across contexts, perspectives and instrument combinations can quickly become prohibitive. Yet a more selective approach can leave analysis highly vulnerable to contingent design features or biases on the part of the analysts themselves.

It is in this regard again, that the emphasis in MCM comes to the fore, of balanced attention across a diversity of specialist stakeholder perspectives. In ways described below, MCM attends in detail not only to quantitative orderings of relative merits, but also to detailed qualitative uncertainties, variabilities and justifications, as elicited from stakeholders. To the extent that cross-dependencies between instruments come to light in these most salient respects, then the otherwise paralysing complexity is reduced. In the present exercise, analysts were especially attendant to this aspect throughout the appraisal interviews. At the end of each interview, interviewees were specifically asked to identify (in light of the detailed appraisal they had just undertaken) the most important negative and positive interactions between different instruments. It is on this basis, that the study discusses some of the more important kinds of interaction between instruments that come to light across different stakeholder perspectives.

## 4 Method for participatory comparative appraisal

Six quality criteria were derived in Section 2, to address the challenge highlighted in Borrás and Edquist (2013) for prospective appraisal of innovation policy instrument mixes. This section describes how the present novel application of MCM aims to respect these criteria, in appraising alternative policy options for promoting AMR diagnostic innovation across six European countries.

**Fig. 1:**
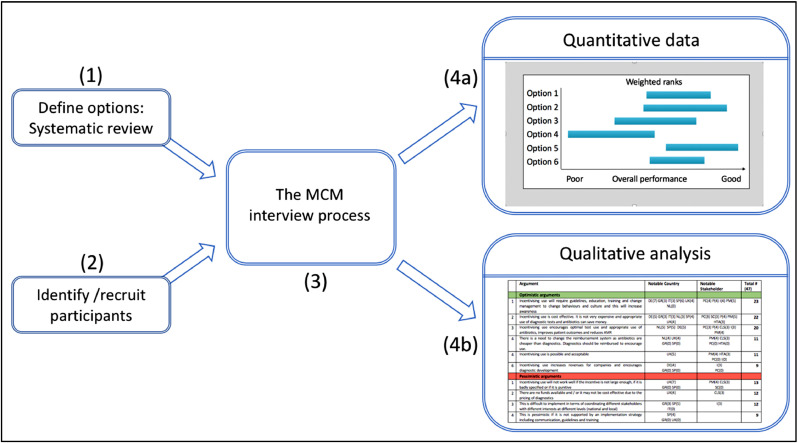
The MCM process from interview preparation to analysis.

MCM is an interactive method for exploring complex strategic and policy issues that is designed to capture different specialist stakeholder perspectives, and their respective rationales for the ‘best’ courses of action. The MCM process has been described in detail elsewhere (Stirling and Mayer, 2001; Stirling et al., 2007; Bellamy et al., 2013) and so only an outline is presented here. The MCM process employed in this study is illustrated in Fig. 1, with the remainder of this section describing the process displayed. Prior to MCM interviews, (1) an appropriate array of relevant options needs to be defined, and (2) interviewees need to be selected, ensuring the inclusion of a relevant plurality of perspectives. At interview, (3) the standard MCM process allows individual interviewees to appraise options according to their chosen criteria, involving in turn (a) systematic development of appraisal criteria; (b) consideration of relevant uncertainties and conditionalities; (c) determination of relative subjective priorities to attach to different criteria; (d) attention to any relevant additional or varied options and finally (e) consideration for the validity of resulting overall orderings of options.

This approach allows in-depth analysis of many different kinds of dimensions – including uncertainties in relation to individual options, issues or perspectives; ambiguities across different perspectives in all these regards; and contrasting rankings under divergent perspectives or across different national or other settings. A novel comparative analysis method is added here to the established MCM approach, drawing on and synthesising the quantitative (4a) and qualitative (4b) data from interviews. Together these allow clearer insights into the selection and implementation of prospective policy instruments and instrument mix appraisal.

### 4.1 Selecting policy options

To define a broad portfolio of policy options for appraisal at the MCM interviews, a two-stage process was followed. In the first stage an exhaustive list of policy instruments to promote innovation in the AMR field was identified. A literature review was undertaken based on sources published between 2004 and 2016. This included policy documents, and academic literature related to AMR and diagnostics.

These were collected through a systematic search, which identified relevant source documents – namely those policy reports or academic articles that discuss diagnostic innovation and AMR, authored since 2000. The search terms used to identify these documents were ‘antimicrobial resistance diagnostics’, ‘antibiotic resistance diagnostics’, and ‘AMR diagnostics’. The results were augmented by AMR strategy documents from prominent bodies developing policy options (e.g. WHO, OECD, and EU). The aim was to collect a wide range of policy types focusing on a particular policy goal: The development and use of diagnostic tests to manage AMR. The search generated a set of 54 relevant source documents with the vast majority published within the last seven years (28 were published in the years 2015–2016, a further 20 between 2010 and 2014, while just five pre-date 2010) (See supplementary materials: Annex A for the full list of references). This is in keeping with the authors’ understanding that AMR has been recognised only recently at the policy level as an emerging global challenge.

The second stage involved classifying the instruments identified in stage one into families of policy instruments with the resulting options initially classified into ten categories, which themselves were classified as Science-Push models (three categories), Market-Pull models (seven categories) and an additional ‘other’ category. These were further reduced to six families of policy instruments, which address a broad range of needs across the innovation system, encompassing the different stages of the innovation process, various mechanisms of action and focusing on a range of actors. These six became the six policy options used in the MCM interviews with stakeholders. Table 1 describes how options were selected and provides a summary of their relative occurance in the literature review (see Annex A). Table 2 sets out the final six options with definitions and details that differentiate them from each other, as shared with interviewees.

**Table 1:** Deriving policy options from a review of the empirical literature on diagnostic innovation relevant to AMR.

	**Selected policy options – for discussion at interview:**						**Other options – not selected for discussion:**	
	**Fund R&D**	**Make pathways**	**Protected markets**	**Enhance revenues**	**Incentivise use**	**Government provides**	**Global / overarching / other issues**	**Communication & dissemination**
**Total times mentioned**	35/54	37/54	3/54	36/54	25/54	3/54	29/54	27/54
**Notes**	Included to represent ‘push’ policies to address market failure	Included to represent co-ordination policies to engage key stakeholders	Included to provide an assessment of the ‘status quo’ and existing IP protection to stimulate R&D.	Included to represent supplier focused policies to address market failures	Included to represent demand focused ‘pull’ policies to address market failures	Included to widen the scope of the options provided	Excluded in order to focus on national level options	Excluded as these themes were considered an essential part of other measures

**Table 2:** Policy options organised by key features.

**Options**	**Description**	**Stage in pathway**	**Mechanism**	**Role of government / industry**	**Actors of focus**
**Enhance revenues**	Government encourages firms to participate in the diagnostics market by increasing financial rewards	Downstream	Pulls suppliers towards the market with incentives	Government and private industry reliant	Diagnostics firms
**Fund R&D**	Government encourages diagnostic innovation by providing researchers with more funding for R&D	Upstream	Pushes new technology towards the market	Government reliant	Public and private researchers, diagnostics firms
**Make pathways**	Government coordinates stakeholders to provide help for firms seeking to bring new tests to market	Upstream	Signals to suppliers the needs of the market	Government and private industry reliant	Diagnostics firms
**Government provision**	Government leads R&D and clinical testing to ensure optimal test use in their healthcare system	Upstream / Downstream	State provision of the required goods and services	Government reliant	Healthcare systems
**Incentivise use**	Healthcare providers create incentives and remove disincentives to encourage better use of tests	Downstream	Encourages demand to grow by incentivising use	Healthcare system reliant	Clinical users
**Protected markets**	New tests are developed based on market demand and established international intellectual property protection regimes	Downstream	Pulls suppliers towards the market with incentives	Private industry reliant	Diagnostics firms

### 4.2 MCM interviewees

Different countries display contrasting needs and contexts with regard to innovation policy for addressing AMR. In order to present results of relevance beyond particular national settings, this study therefore aims to span a diverse range of circumstances, whilst at the same time interrogating and reporting specific salient sites of investigation in their own terms. Among the explicit dimensions of this diversity, countries were selected in order to display variation in AMR prevalence (which correlates with antibiotic use), as well as in diagnostics market size. For AMR prevalence, although there is variation depending on the bacterial species and antimicrobial group, overall Germany and the Netherlands have low rates, while Italy and Greece have higher rates and the UK and Spain have more moderate rates (ECDC, 2017). In terms of economic size, Germany, Italy, Spain and the UK are amongst the largest European economies, offering large markets for diagnostics developers, with the Netherlands and Greece representing smaller economies offering smaller markets. Table 3 summarises some relevant contextual details of the six European countries studied.

**Table 3:** Selected details for six European countries.

**Country**	**Germany**	**Greece**	**Italy**	**Netherlands**	**Spain**	**UK**	**Source**
**Population, total**	81,844,000 (2011)	11,237,000 (2008)	60,900,000 (2012)	16,900,000 (2014)	46,158,000 (2008)	64,500,000 (2014)	HiT reports*
**GDP, PPP (billion)**	$3,307 (2012)	€269 (2008)	$2,017 (2012)	$803 (2014)	$1,461 (2008)	$2,525 (2014)	HiT reports*
**GDP per capita PPP**	$40,725 (2012)	€21,300 (2008)	$33,110	$45,691 (2011)	$31,586	$39,137	HiT reports*
**Total health expenditure PPP per capita**	$4,495 (2011)76.5% public, 23.5% private	$2,727 (2007)60.3% public, 39.7% private	$3,040.1 (2012) 80.8% public, 19.2% private	$5,601 (2013)	$2,671 (2007)71.8% public	$3,311 (2013)(83.5% public)	HiT reports*
**Total health expenditure as% of GDP**	11.3	9.6	9.2	12.9	8.5	9.1	HiT reports*
**Health system**	Health services are funded by mandatory health insurance, 85% statutory health insurance and 11% substitutive private health insurance.	There is a mix of public and private funding.Public funding is from social insurance and private is mainly from out-of-pocket payments.	National health service with universal coverage, mainly financed by national and regional taxes.	Bismarckian social health insurance hybrid with regulated competition between insurers.	The (devolved) health system provides universal coverage funded from taxes and predominantly operates within the public sector.	Regionally devolvednational health service, mainly funded through general taxation, with the remainder coming from private medical insurance and out-of-pocket payments.	HiT reports*
AMR ranking** (1=lowest AMR)	2	6	5	1	4	3	(Goossens et al., 2005 cited by O'Neill, 2015)
**Total Molecular Diagnostics Market:% Revenue by Region, Western Europe, 2015**	27.2	-	17.1	2.5	12.1	11	(Frost and Sullivan, 2017)
Funding ranking*** (1=most projects)	2	5	4	3	4	1	(Kelly et al., 2016)

*HiT reports:, Germany (Busse et al., 2014)**, Italy** (Ferré et al., 2014)**, Netherlands** (Kroneman et al., 2016)**, Spain** (García-Armesto et al., 2010)**, UK** (Cylus et al., 2015).

**by Penicillin non-susceptible S. pneumonia (%) (2001).

***by total number of AMR diagnostic projects per country by priority topic funded at national level.

In order to assess policy options, interviewees were selected (i) to help analysts understand key country specificities and (ii) to span a diversity of stakeholder groups to bring understanding and experience from across each national health system.

In each country, interviews were conducted with individuals from seven distinguishable stakeholder groups. These stakeholder groups were addressed in order to garner a salient plurality of perspectives on the diversity of circumstances noted above. Specific choices were based on understandings from prior research on diagnostics innovation (e.g. Hopkins and Nightingale, 2006). Groups included: primary care clinicians, secondary care clinicians, clinical laboratory scientists, pharmacists, industry executives, policy makers, and health technology assessors, including health insurers/ payors (henceforth HTAs). A majority of the interviewees (36/47) were selected because of their individual involvement in national expert groups on AMR.

While good coverage was achieved for the countries and stakeholder perspectives listed above; other identifiable kinds of perspective were not addressed in such balanced ways. For instance, much less than half of the sample were female (10/47); and the patient voice is missing from the study, as prominently engaged representatives of this group could only be identified and interviewed in one country. Table 4 summarises stakeholder participation in each country. Interviewees that did not have experience of national expert groups on AMR are indicated with a *.

**Table 4:** Interviewees by country, stakeholder group and experience.

**Stakeholder group**	**Germany**	**Greece**	**Italy**	**Netherlands**	**Spain**	**United Kingdom**	**Total**
**HTA**	1	1	1	1*	1	1	6
**Industry**	1	1	1*	1*	2	1	7
**Clinical lab scientist**	1	1*	2	1	1*	1	7
**Pharmacist**	1	1	1	1	1*	1	6
**Policy maker**	2 (1*)	1	1	1	2	1	8
**Primary care clinician**	1	1*	2	1	1*	1	7
**Secondary care clinician (infection)**	1	1*	1	1	1	1	6
**Total**	8	7	9	7	9	7	47

*Interviewee not on a national AMR panel.

Each interview was conducted face-to-face by two team members in overlapping combinations to help ensure consistency in application of the interview protocol (FB/MH, FB/JMF, JC/FB, JC/MH and JC/MH + SA ). Additional language translation was provided as needed in Greece, Italy and Spain, mainly by authors (SA for Greece, JMF for Spain) or exceptionally by local PhD students (see acknowledgements). Interviews lasted from 45 min to 2½ hours. All interviews were conducted between March 2017 and March 2018.

The analysis presented here is based on 47 of 50 interviews undertaken. MCM is a cognitively demanding approach, consequently, three interviewees were not able to provide full quantitative MCM assessments at interview. As a result, quantitative and qualitative data from these interviews have been excluded from the analysis. Interviewees were free to appraise policy instruments in any order, thus reducing the scope for some instruments to be less carefully appraised due to undue influence from the protocol structure.

A possible query that arises at this point, concerns how this array of 47 interviews spanning seven identified perspectives across six countries, relates to received ideas in probabilistic sampling? It was already discussed in Section 2, that prevailing conditions of uncertainty and ambiguity (that the MCM process specifically seeks to address and reveal), render it difficult to justify any unitary objective aims or claims around ‘statistical representativeness’ (Stirling, 2010; O'Neill, 2001). In keeping with understandings that are well established both in quantitative (Rothman et al., 2013) and in wider social research (Tracy, 2013)(Somekh and Lewin, 2005), such notions depend on prior confidence that the dimensionalities, definitions and partitionings are complete and definitive concerning what might constitute salient categories (such as ‘parent populations’; ‘causal mechanisms’; or ‘frequency distributions’) (Kahneman and Tversky, 1974). In MCM (Stirling, 1997), as in other research methods, such as Q method (Stephenson, 1953), it is precisely these issues of framing and objectivity that are under scrutiny. So, statistical sampling methods are better understood as being *dependant on* (rather than *prior to*) the kinds of phenomena examined in MCM (Coburn et al., 2019).

In these terms, the recruitment of perspectives for elicitation in MCM interviews might most appropriately be held to be subject to general disciplines of ‘scientific inference’ rather than ‘statistical inference’ (Mitchell, 1983). With each elicitation in its context also a unit of analysis in its own right (Small, 2009), the appropriate form of reasoning is in this regard more akin to ‘case study logic’ than to ‘sampling logic’ (Yin, 2009). Relying more on ‘non-probabilistic methods’ (Parker et al., 1998), this raises considerations of ‘validity’ more than representativeness (Mitchell, 1983), ‘theoretical sampling’ more than statistical sampling (Silverman, 1989), and ‘potential for learning’ more than calculative generalisation (Stake, 1994). In interpreting MCM results as outcomes of deliberative policy appraisal involving inherently subjective framings (Davies et al., 2003) – qualities of ‘inclusiveness’ (O'Neill, 2001), ‘legitimacy’ (Chilvers, 2009) and ‘transparency’ (Stirling, 2003) are more important than what has been termed the “*false essentialism”* of ‘statistical representativeness’ (Smith and Wales, 2000).

It is in the above terms that the 47 in-depth, systematic interviews undertaken for the purpose of this study, might reasonably be judged to be an appropriate number in relation to the style and purpose of this analysis: mapping salient parameters of contrasting policy options and criteria, rather than seeking to generalise perspectives (Stephenson, 1953). Either way, since what is under scrutiny in MCM are features of diverse framings in social discourse more than contingent psychologies, the most pertinent focus for statistical analysis would lie less in the number of participants, than in the many hundreds of data points (both scores and qualitative statements) associated with policy options and criteria. In any case, with the value of results lying in the learning they enable, the adequacy or otherwise of the particular elicited perspectives is better assessed in relation to the qualities of results (as set out in this article but also in the supplementary materials: Annex C and D, intended to inform policy implementation), than in the quantities of inputs.

### 4.3 **The MCM interview process**

MCM software is used in this study to facilitate a structured interaction with stakeholders for the systematic appraisal of policy options at interview. Alternative options (i.e. different families of policy instruments) are presented to the interviewees as possible actions to achieve a ‘focal goal’, i.e. to promote the development and use of diagnostics to manage AMR through reduced antibiotic consumption. In MCM, participants are encouraged to think how different options may perform, taking into account complexities, uncertainties and ambiguities, and allowing themselves to move away from any notionally single ‘best answer’. MCM aims instead at ‘plural and conditional’ inputs to policy making (Stirling, 2006, Stirling, 2008b, Stirling, 2010). To do so, the interview process aims at unpacking these through different steps in the appraisal. After reviewing policy options, participants select a set of criteria that they think most closely affect the ability of each option to contribute to the focal goal (articulating *conditionalities*). The criteria selected by interviewees in this study are available in the supplementary materials: Annex C. Under each criterion, interviewees express their appraisal of each policy option by assigning, an optimistic and a pessimistic score (to account for any *uncertainty* or *conditionality,* for example, related to mode of policy implementation), which is entailed with a discussion that supports these scores. After appraising each option from the perspective of each criterion, interviewees are able to compose an aggregated chart which accounts for the quantitative assessment of all the policy options under all criteria, with each criterion weighted by importance (see Fig. 1:(4a) Weighted ranks chart). The chart is shown to interviewees, who can iterate their appraisals as desired (together with supporting qualitative justifications) until they approve the final output of the discussion. At this stage, the interview moves on to elicit views on potential complementarities or conflicts between policy options, to inform an understanding of appropriate mixes.

### 4.4 **Analysis of the MCM quantitative and qualitative data**

**Fig. 2:**
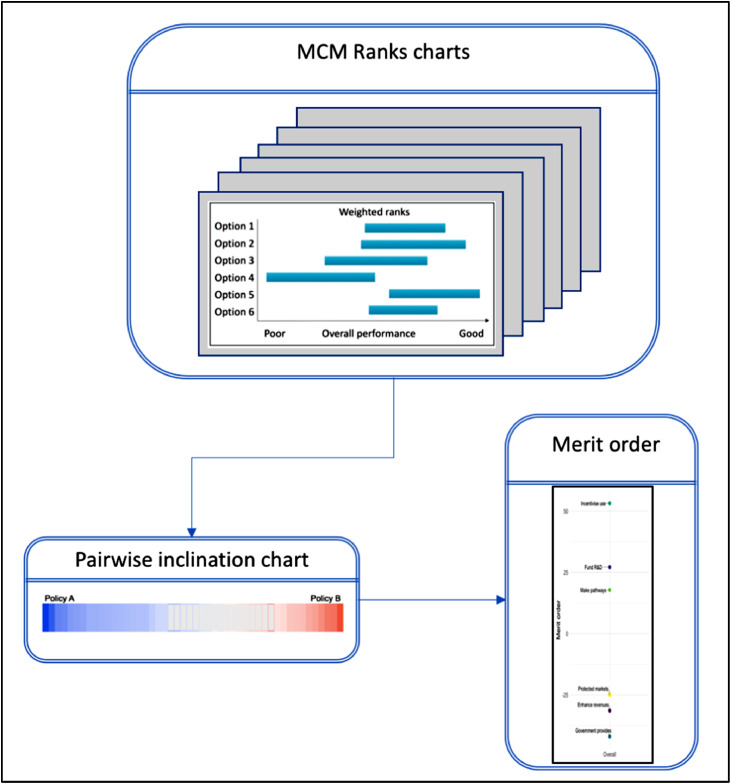
Analysis of quantitative data from MCM.

Thus far, it has been argued that the MCM process provides an approach that addresses the six criteria in Section 2, enabling the gathering of a rich array of quantitative and qualitative data. New methodological improvements to MCM that facilitate the analysis on both the qualitative (see Fig. 1(4b)) and quantitative front (see Fig. 2) are presented here as a way to enhance prospective appraisal of policy mixes. For reasons of space, the technicalities behind these improvements are detailed separately (see MCM manual (Coburn et al., 2019)).

**Fig. 3:**
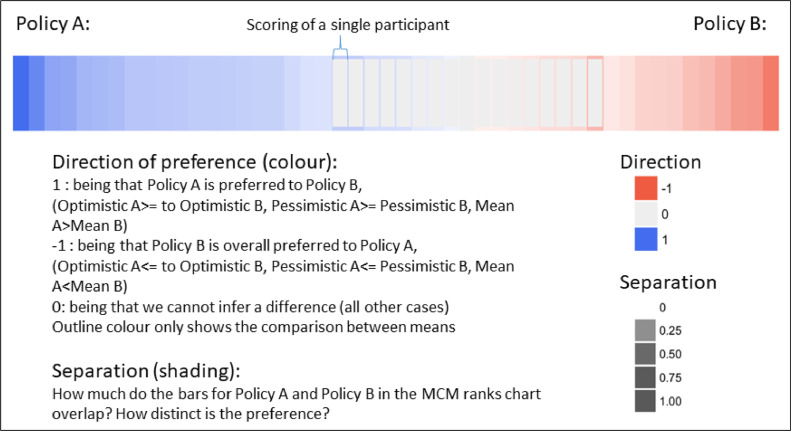
Example of a pairwise inclination chart (47 interviewees) and how it is constructed using direction of preference and separation.

Two new forms of data visualisation and analysis for MCM are applied here for the first time. These are what we refer to as the ***pairwise inclination chart*** and the ***merit order***. As Fig. 2 indicates, the pairwise inclination chart uses the optimistic and pessimistic scores and the difference between them (i.e. the length of the bar) from the final ranks charts produced by each MCM interviewee to compare their appraisal of each possible pairing of the core policy options that they have appraised (i.e. six policy options yields 15 possible pairings for comparative purposes from each interviewee). Subsequently, the outcomes from the analysis of all possible pairings are used to generate an overall merit order, distinguishing higher and lower performing options. In Fig. 3 each pairwise comparison from each interviewee's perspective is accorded equal focus (i.e. fairly engaging with a plurality of perspectives). The figure represents the appraisals of all interviewees as a series of cells, each representing one interviewee's assessment of the relative performance of one option over the other. The outcome is represented by the colour: superior performance of policy A is indicated in blue, and superiority of policy B is indicated in red. The degree of uncertainty expressed by interviewees is indicated through the degree of shading, with deeper shading indicating less uncertainty (for a full explanation of the chart and underlying calculations please refer to pages 105–111 in Coburn et al., 2019). Uncoloured (grey) cells show instances where there is no discernible inclination to be inferred from the optimistic and pessimistic scoring of either option in the pairwise comparison. The chart in its entirety provides a clear indication of the proportion of interviewees more inclined to view one policy option as superior in performance than another while also communicating the general uncertainties expressed by stakeholders. For example, in Fig. 3, the greater quantity of blue shading indicates that interviewees are more inclined towards policy A than policy B overall, while the light shading and grey cells show the uncertainties expressed by participants.

Merit orders are derived by summing scores across participants for each policy option. These participants’ merit scores combine separation (between 0 and 1) with the direction of preference (+ or − sign). For a full description of how merit scores and merit orders are computed see MCM manual pp.105-111 (Coburn et al., 2019). They provide a synthesis of pairwise comparisons, where policy options higher in the order have been appraised more positively overall. The separation between the options in the merit order indicates the margin by which an option can be said to be favoured compared to the adjacent policy options. Merit orders can be generated for all interviewees, to provide an overall view, or for subsets of interviewees to tease out differences in perspectives, thus providing the ability to understand the role of these perspectives in determining the overall merit order.

Yet, a deep understanding of context, conditionality and uncertainty cannot be obtained without the qualitative data accompanying quantitative scores. In keeping with the aim to provide a fair level of engagement between all interviewees, the qualitative comments have also been analysed in a systematic way, pooling comments by policy option, thematically grouping similar issues (along with conditionality and expressions of uncertainty) and tallying these according to whether they were a part of optimistic or pessimistic appraisals (a detailed explanation of the extraction and grouping process can be found in the MCM manual pp. 94–104 (Coburn et al., 2019)). The qualitative data can be used to derive detailed insights into the expectations of interviewees for particular policy options. For reasons of space, only those themes that recurred in more than 10% of interviews are displayed in the tables provided in Section 5.

## 5 Empirical results

Results are reported from 47 interviews across seven stakeholder groups in six countries, based on their appraisal of the same core set of policy options. The options are referred to by their names, e.g. ‘government provides’ and ‘incentivise use’, following the definitions set out in Table 2.

This section addresses the second and third steps of the process for prospective policy instrument mixes set out by Borrás and Edquist (2013), namely the design and customisation of instruments and the design of instrument mixes. The section is divided into three subsections. The first offers an aggregated view of the quantitative data in order to provide suitably-qualified indications of which options might be expected to perform most favourably across a range of European contexts. These are accompanied by analysis of pooled qualitative comments from all countries and stakeholder groups revealing commonly anticipated advantages and disadvantages for each policy option. Together, this analysis contributes a key basis for understanding how policy options can be optimised to address local needs and the conditions under which these are expected to perform well or poorly. In the second subsection, the disaggregation of appraisals by stakeholder group and country provides further insights concerning the varying suitabilities of different policy options to address AMR in particular national settings as seen from contrasting perspectives. In the third subsection, the potential is examined for policy options to be complementary or to conflict with each other.

MCM interviewees are encouraged to add and appraise additional policy options where they feel further options ought to be considered alongside the core options. While additions cannot be appraised by other interviewees, these may offer further insights into the comprehensiveness of the core options used, as well as suggesting other possibilities for further enquiry. The additional options suggested by interviewees are listed in the supplementary materials: Annex C.

### 5.1 Aggregated analysis

**Fig. 4:**
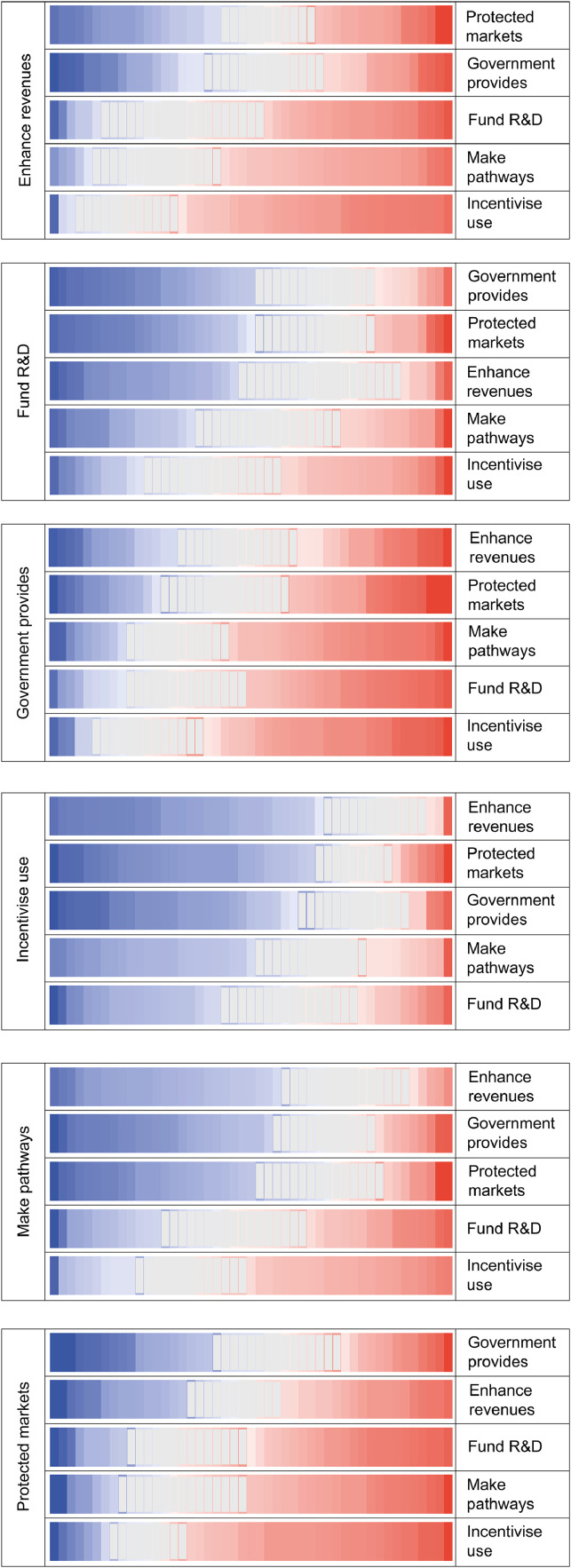
Pairwise comparisons of policy options from all interviewees (see Fig. 3 for an example showing how these charts were constructed using direction of preference and separation).

Fig. 4 displays the results from the pairwise comparisons from all interviewees (i.e. 15 policy option pairing x 47 interviewees). In each panel (A-F) of Fig. 4 a given policy option is compared with all other policy options to reveal interviewees’ inclinations on the expected relative performance of each policy option. The key to interpreting the visualisation of data in Fig. 4 is provided in Fig. 3.

As indicated by the shading conventions explained in the diagram key (in Fig. 3), Fig. 4's Panel D shows that ‘incentivise use’ is generally more favourably appraised (intensity of blue shading on the left of the diagram) by more interviewees (numbers of cells), than other policy options (inclinations for which are indicated by red shading on the right of the diagram). The degree of ambiguity with which this remains the case is conveyed by considering the relative number of interviewees who display the opposite view (red) and by the intensity of shading in both (blue and red) views - (less strong relative orderings are indicated by less intense colours). It is quite clear across interviewees generally that they expect ‘incentivise use’ to perform better than ‘government provides’ and ‘protected markets’. Conversely, Panel C shows that ‘government provides’ is the policy option that tends generally to perform least well when compared with all others. However, it is inherent to the complexities of appraisal reflected in MCM, that even this least favoured option has a relatively small number of supporters for whom it performs comparatively strongly, particularly in comparison to ‘enhance revenues’ and ‘protected markets’. Specific reasons behind this are documented in the qualitative results.

With ‘incentivise use’ tending to be the most favoured option overall, other highly favoured options are (in declining order of support) ‘fund R&D’ (shown in Panel B) and ‘make pathways’ (Panel E). An overall majority of interviewees tend to find these options more favourable than others. However, the relatively lighter shading (both of red and blue) indicates that there tends to be greater uncertainty across interviewees concerning the potential performance of ‘make pathways’. Finally, this pairwise comparison suggests that ‘enhance revenues’ (Panel A) and ‘protected markets’ (Panel F) tend to be somewhat less favoured options. Those pairs of options that tend to elicit more divergent appraisals from interviewees can be seen to generate a greater proportion of stronger shading in both red and blue.

**Fig. 5:**
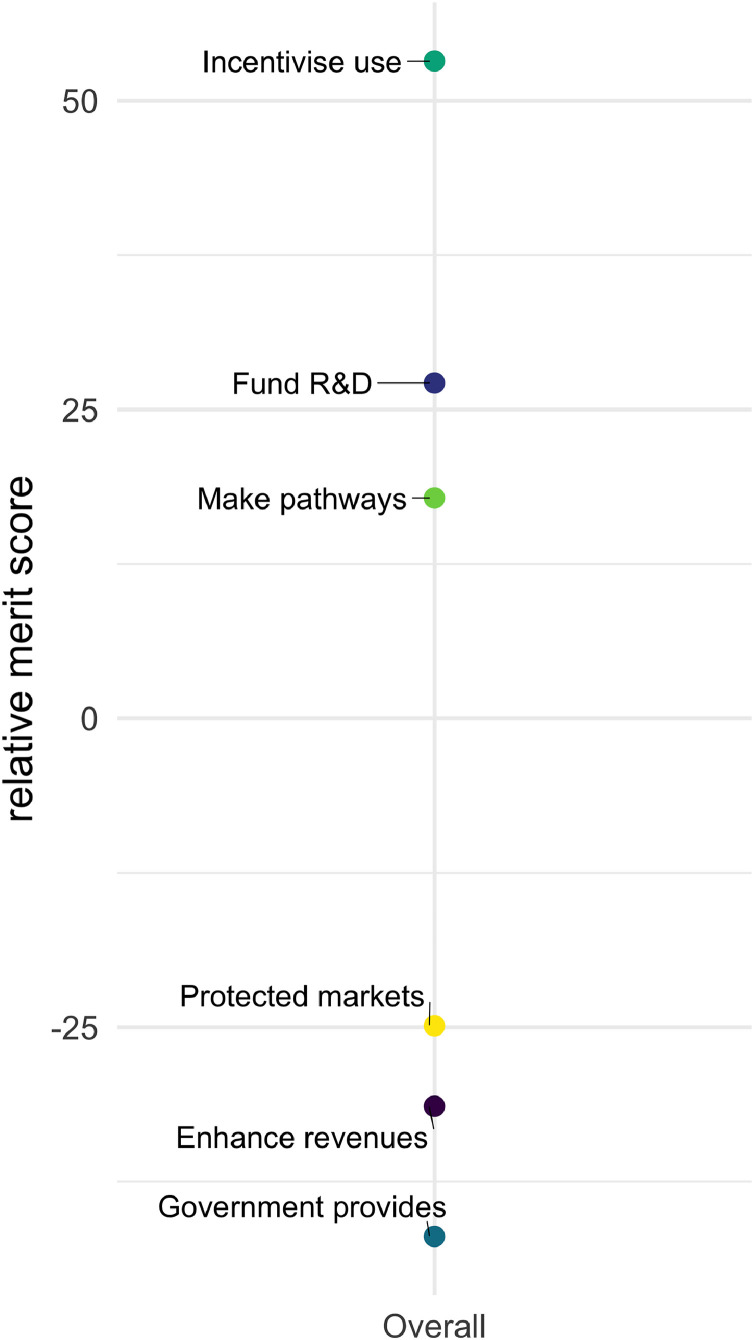
Merit order of six policy options (based on 47 interviewee appraisals).

Across all pairwise comparisons, a highly general relative ordering for all options can be expressed in the form of a merit order (see MCM manual (Coburn et al., 2019). Since the merit order averages across so many contexts, criteria and perspectives, it should be treated with great caution – with the aggregate picture complemented by the patterns evident in disaggregated quantitative and qualitative results (see Section 5.2). However, simply as a broad heuristic the above general pattern is confirmed in Fig. 5, which quite clearly distinguishes between the overall favourability of a group of three ‘top’ (+) and three ‘bottom’ (-) policy options. Again, these results indicate by a considerable margin, that ‘incentivise use’ is the instrument that tends overall to perform best in the widest range of circumstances. This is followed by ‘fund R&D’ and ‘make pathways’. The three options that tend to perform less well are ‘protected markets’ and ‘enhance revenues’, which have broadly similar levels of support. Finally, ‘government provides’ is the option that tends to perform least well.

### 5.2 Analysis by policy option

**Fig. 6:**
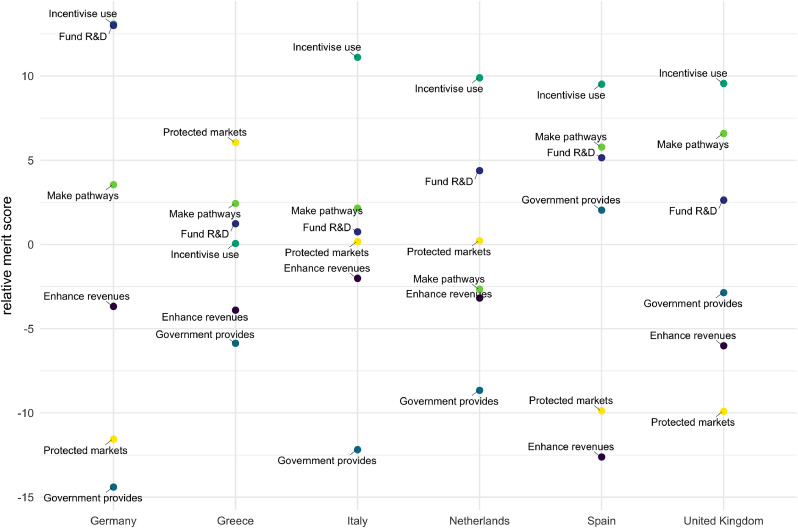
Merit order by country-level perspectives of interviewees.

**Fig. 7:**
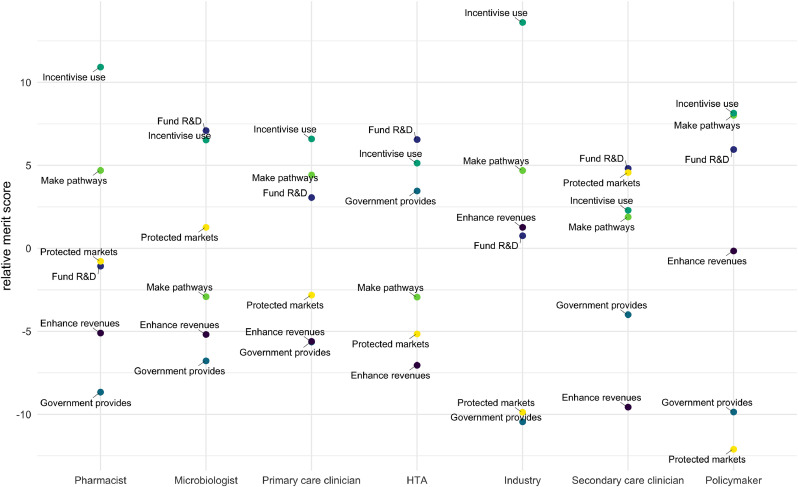
Merit order by stakeholder group.

This overall ordering of options can be contextualised by the disaggregated picture for different settings and perspectives (e.g. Figs. 6 and 7 discussed in the following section) as well as the qualitative responses that interviewees were asked to provide as commentary during the course of their appraisals of the different policy instruments. This latter rich body of information arises from each interviewee being frequently prompted to give reasons, caveats or other comments concerning different detailed aspects of their appraisals. These qualitative responses are discussed in the following section.

Key features of the corpus of qualitative responses gathered from the 47 interviewees are summarised in Table 5, Table 6, Table 7, Table 8, Table 9, Table 10. These may help inform policy makers of many considerations helpful for the design of policy instruments or their modes of implementation. For example, the tables indicate which kinds of more commonly anticipatable problems might arise in different cases and suggest specific options that may be more or less suited in different contexts.

**Table 5:** Optimistic and pessimistic qualitative comments about 'incentivise use'.

	**Argument**	**Notable Country**	**Notable** **Stakeholder**	**Total** **# (47)**
	**Optimistic arguments**			
1	Incentivising use will require guidelines, education, training and change management to change behaviours and culture and this will increase awareness	DE(7) GR(3) IT(3) SP(6) UK(4) NL(0)	PC(4) P(4) I(4) PM(5)	**23**
2	Incentivising use is cost effective. It is not very expensive and appropriate use of diagnostic tests and antibiotics can save money.	DE(5) GR(3) IT(3) NL(3) SP(4) UK(4)	PC(3) SC(3) P(4) PM(5) HTA(3)	**22**
3	Incentivising use encourages optimal test use and appropriate use of antibiotics, improves patient outcomes and reduces AMR	NL(5)SP(5)DE(5)	PC(3) P(4) CLS(3) I(3) PM(4)	**20**
4	There is a need to change the reimbursement system as antibiotics are cheaper than diagnostics. Diagnostics should be reimbursed to encourage use.	NL(4) UK(4) GR(0) SP(0)	PM(4) CLS(3) PC(0) HTA(0)	**11**
4	Incentivising use is possible and acceptable	UK(5)	PM(4) HTA(3) PC(0) I(0)	**11**
6	Incentivising use increases revenues for companies and encourages diagnostic development	DE(4) GR(0) SP(0)	I(3) PC(0)	**9**
	**Pessimistic arguments**			
1	Incentivising use will not work well if the incentive is not large enough, if it is badly specified or if it is punitive	UK(7) GR(0) SP(0)	PM(4) CLS(3) SC(0)	**13**
2	There are no funds available and / or it may not be cost effective due to the pricing of diagnostics	UK(4)	CLS(3)	**12**
3	This is difficult to implement in terms of coordinating different stakeholders with different interests at different levels (national and local)	GR(3) SP(5) IT(0)	I(3)	**12**
4	This is pessimistic if it is not supported by an implementation strategy including communication, guidelines and training	SP(4) GR(0) UK(0)		**9**

PC (Primary care), SC (Secondary care), P (Pharmacist), CLS (Clinical lab scientist), I(Industry), HTA, PM (Policy maker).

**Table 6:** Optimistic and pessimistic qualitative comments about 'fund R&D'.

	**Argument**	**Notable Country**	**Notable** **Stakeholder**	**Total #** **(47)**
	**Optimistic arguments**			
1	Collaboration between public and private are important	DE(4) IT(3) SP(3)	HTA(4) PM(4) P(0)	**15**
1	Particularly clinical utility and cost effectiveness research to provide evidence	DE(4) SP(3) UK(4)	PC(3) I(3)	**15**
3	It's important, acceptable and necessary	GR(3) NL(3) SP(4) UK(3) DE(0)	PM(4) CLS(4) SC(0)	**14**
4	Funding R&D is feasible	DE(3) IT(4) UK(3) NL(0)	CLS(3) I(0)	**12**
5	Funding R&D generates knowledge and encourages diagnostic innovation and development of novel tests	DE(3) NL(4) IT(0) UK(0)	HTA(0)	**10**
6	Funding R&D is cost effective	NL(7) UK(7) DE(0) GR(0) IT(0)	CLS(0)	**8**
	**Pessimistic arguments**			
1	Funding R&D may not be successful	NL(3) SP(3) GR(0) IT(0)	HTA(0)	**9**
2	There is not enough money available for funding R&D	DE(0) UK(0)	HTA(0) P(0)	**7**
2	There may be problems with bureaucracy	NL(0)	PC(0) I(0)	**7**
2	If funding does not include clinical validation	DE(4) UK(3) GR(0) IT(0) NL(0) SP(0)	PC(0)	**7**

PC (Primary care), SC (Secondary care), P (Pharmacist), CLS (Clinical lab scientist), I(Industry), HTA, PM (Policy maker).

**Table 7:** Optimistic and pessimistic qualitative comments about 'make pathways'.

	**Argument**	**Notable Country**	**Notable** **Stakeholder**	**Total # (47)**
	**Optimistic arguments**			
1	Making pathways could coordinate different stakeholders and create consensus between the stakeholders about what is needed	DE(6) GR(6) IT(4) NL(3) SP(5) UK(3)	PC(3) P(5) CLS(4) I(5) PM(6)	**27**
2	Making pathways is cost effective. It is not very expensive and improving coordination usually saves money	SP(4) UK(5) IT(0)	SC(3) PM(5) CLS(0)	**14**
2	Pathways are already being made. It is acceptable and feasible.	DE(3) IT(3) UK(4)	CLS(3) PM(4) HTA(3)	**14**
4	Making pathways is good for getting tests to market, getting them into use, and for patient outcomes	SP(3) UK(3) GR(0) IT(0)	PC(0)	**8**
5	Clear evidence-based guidelines are needed and making pathways also helps to clarify what kind of evidence is needed.	DE(3) NL(3) GR(0) SP(0) UK(0)	CLS(0)	**7**
	**Pessimistic arguments**			
1	Coordinating a complex system with multiple stakeholders is difficult	GR(4) IT(6) NL(5) SP(4)	PC(3) SC(3) P(4) I(4) PM(4)	**22**
2	This option is not needed as adequate pathways already exist	GR(0)	HTA(0)	**8**
3	Making pathways is not cost effective, particularly if there is no impact	GR(0) IT(0) NL(0)	P(0) CLS(0) I(0)	**6**

PC (Primary care), SC (Secondary care), P (Pharmacist), CLS (Clinical lab scientist), I(Industry), HTA, PM (Policy maker).

**Table 8:** Optimistic and pessimistic qualitative comments about 'enhance revenues'.

	**Argument**	**Notable Country**	**Notable** **Stakeholder**	**Total# (47)**
	**Optimistic arguments**			
1	Enhancing revenues encourages more companies to innovate and develop diagnostic tests	DE(3) NL(5) SP(3) UK(3)	CLS(4) I(3) PM(4) HTA(4) PC(0)	**17**
2	Enhancing revenues is feasible and realistic	DE(4) UK(3) GR(0)	HTA(4) PC(0) SC(0)	**12**
3	Collaboration and communication between public and private are important	IT(4) DE(0) SP(0)	SC(0) PM(0)	**8**
	**Pessimistic arguments**			
1	Enhancing revenues is expensive and not cost effective	NL(4) SP(5) UK(6) DE(0) IT(0)	SC(4) I(3) PM(4) CLS(0)	**17**
2	Government cannot / should not provide additional funding to companies	SP(3)	PC(3) CLS(3)	**13**
3	There is a risk that the focus for firms is on maximising profits and not on societal benefit	DE(3) GR(4) NL(3) IT(0) SP(0) UK(0)	SC(3) P(3) PM(0) HTA(0)	**10**
4	There are uncertainties about patient needs and resistance, quality of evidence and product, clinical utility, and how to value diagnostics	DE(3) IT(0) NL(0)	I(3) PC(0) SC(0)	**8**

PC (Primary care), SC (Secondary care), P (Pharmacist), CLS (Clinical lab scientist), I(Industry), HTA, PM (Policy maker).

**Table 9:** Optimistic and pessimistic qualitative comments about 'protected markets'.

	**Argument**	**Notable Country**	**Notable** **Stakeholder**	**Total #** **(47)**
	**Optimistic arguments**			
1	Protecting markets encourages diagnostic innovation based on needs by making the diagnostics market profitable and this improves patient management	DE(5) IT(4) NL(4) SP(4)	PC(4) P(3) CLS(4) PM(5)	**20**
2	Protecting markets does not cost much	GR(3) UK(3) DE(0) NL(0) SP(0)	CLS(0) I(0)	**7**
3	Protecting markets is necessary but not sufficient on its own. It is a prerequisite.	DE(0) NL(0) SP(0)	PC(0) I(0) HTA(0)	**5**
3	This is already internationally established and used everywhere in the world. The rules are already there, the principles are already there.	IT(0) UK(0)	P(0) CLS(0)	**5**
	**Pessimistic arguments**			
1	Protecting markets costs money and doesn't add much to the policy portfolio.This could mean too much money being paid to industry.	IT(3) UK(4)	SC(3) PM(6) CLS(0) I(0)	**12**
2	Protecting markets is not working well and it is not going to work well	UK(6) DE(0) GR(0) IT(0) NL(0) UK(0)	SC(0)	**7**
2	Protecting markets is about money and not sustainability, improving evidence, patient access, or preserving effectiveness	IT(4) DE(0) GR(0) UK(0)	CLS(0)	**7**

PC (Primary care), SC (Secondary care), P (Pharmacist), CLS (Clinical lab scientist), I(Industry), HTA, PM (Policy maker).

**Table 10:** Optimistic and pessimistic qualitative comments about 'government provides'.

	**Argument**	**Notable Country**	**Notable Stakeholder**	**Total # (47)**
	**Optimistic arguments**			
1	Government provision could be cost effective if governments are in control of the whole process and can take the lead on pricing and reimbursement	GR(3) UK(3)NL(0)	CLS(0)	**10**
2	This could happen if there is a national crisis, a change in political will, a market failure or for particular cases	UK(4)GR(0) IT(0)	PM(4)P(0) HTA(0)	**8**
3	Optimistic if the government leads on improving access to laboratories and bacteria, and on improving the quality of clinical testing and evidence	IT(0) SP(0)	PC(0) SC(0)	**7**
	**Pessimistic arguments**			
1	Government provision would be prohibitively expensive, it could be a waste of money and it is not likely to be cost effective	IT(3) NL(4) SP(3) UK(5)	PC(3) SC(3) PM(7)CLS(0)	**19**
2	Government does not have the capacity or capabilities to do this	DE(3) SP(4) UK(3)IT(0)	SC(3) PM(4)	**13**
3	Government provision would be slow	DE(3)GR(0) SP(0)	PC(0)	**8**
4	There is not the infrastructure to do government provision	IT(3)DE(0) NL(0)	PM(3)P(0)	**8**
5	Government provision will not work.It is not a good option.	DE(3) UK(3)GR(0) IT(0) NL(0)	PC(0) SC(0)	**8**

PC (Primary care), SC (Secondary care), P (Pharmacist), CLS (Clinical lab scientist), I(Industry), HTA, PM (Policy maker).

The MCM process cues each interviewee to make as many comments per instrument as they think is appropriate, hence the numbers of responses recorded in the tables varies (although each interviewee's contributions are counted towards each theme only once). The number of occurrences indicated in the tables refers to how many of the 47 interviewees made comments that fall within a given theme. Given that each perspective (country or stakeholder group) is represented by between 6 and 9 independently selected interviewees, where three or more from a single perspective contributed to the same theme this is highlighted in the relevant table as a notable replication of interviewee findings. Similarly, where no interviewee from a given perspective commented on a theme, this is also indicated as notable (although again, due to space, not all of these details can be discussed in this section).

Table 5 shows that almost half of interviewees (23/47) thought that change management, new guidelines and/or communication strategies would be needed to ensure successful implementation of the most favoured option, ‘incentivise use’. Interviewees often suggested that this policy option had some form of financial merit (21/47) and they explicitly indicated it could be beneficial for patients and/or reduce AMR (20/47). There was less consensus regarding the pessimistic views expressed, but a frequent concern was that the incentive selected might not be effective (13/47), with UK interviewees strongly contributing to this point. Another concern was that a sufficiently motivational incentive might not be affordable or cost effective (12/47). Again, this was particularly a concern held by a high proportion of interviewees in the UK and in the clinical lab scientist stakeholder subset. A concern that coordination issues might not be suitably addressed was also raised (9/47), being highlighted most often by interviewees in Spain, and most often by the industry stakeholder subset.

Table 6 shows the most frequent interviewee responses to the second most favoured policy instrument, ‘fund R&D’. This was often expected to work best if it could encourage public-private collaborations (15/47) or when the focus was on providing evidence of the clinical utility or cost effectiveness of tests (15/47). Interviewees frequently said this was an acceptable (14/47) and feasible (12/47) policy option. Pessimistically, some were concerned that R&D funding schemes were not always successful (9/47), a point raised mainly in the Netherlands and Spain. Affordability was also sometimes a concern (7/47). Other concerns were that R&D funding schemes could be too bureaucratic (7/47). Finally, there was concern raised by some UK and German interviewees that R&D funding might not focus sufficiently on the clinical validation of diagnostic testing (7/47).

Table 7 shows that ‘make pathways’ was seen as a useful way to coordinate stakeholders and reach consensus on the tests required to manage AMR (27/47). It was often expected to be cost-effective or at least not too expensive (14/47) and was frequently seen as an acceptable or feasible option (14/47). However, many said that stakeholder coordination was difficult in complex healthcare systems (22/47). This was raised most often as a concern by interviewees in Italy, the Netherlands, Spain and Greece, and it was also particularly a concern for policy makers, industry, and pharmacists. Some thought that this instrument would not be cost-effective (6/47) or that sufficient pathways were already in place and no further actions of this kind were needed (8/47).

Table 8 shows that arguments in favour of ‘enhance revenues’ included that it could encourage firms to develop innovative tests (17/47). This was seen as a feasible option by at least some interviewees across country subsets (12/47). Some viewed ‘enhance revenues’ as more favourable when undertaken with collaboration and/or communication between public and private sectors (8/47), with this view mentioned most often by interviewees in Italy. However, there were frequent concerns that this instrument would be expensive or not cost effective (17/47). This concern was held more often by interviewees in the UK, Netherlands and Spain, and particularly by policy makers, industry and secondary care clinicians. Interviewees often suggested that firms should not be offered additional money to develop tests (13/47). This was most mentioned by interviewees in Spain, by primary care clinicians and by clinical lab scientists. Concerns that ‘enhance revenues’ could lead to profiteering without delivering societal benefit (10/47) were raised by a high proportion of interviewees in Germany, Greece and the Netherlands, and particularly by policy makers and secondary care clinicians.

The ‘protected markets’ option provided interviewees with an opportunity to discuss the status quo regarding the current provision of intellectual property protection as an incentive to bring diagnostic tests to market. As Table 9 shows, this was seen to be beneficial by many interviewees as it encourages innovation (20/47) and some interviewees from Greece, Germany and the UK viewed this as a relatively inexpensive policy (7/47). Protecting markets was seen by some as necessary, but not sufficient to address the challenge of diagnostic innovation to manage AMR (5/47). A few also noted that it was already well established (5/47). Some saw ‘protected markets’ as too costly for the benefits it provided (12/47), with this view being most often mentioned in the UK and Italy, and by policy makers and secondary care clinicians. Some commented that ‘protected markets’ was not working well in the context of diagnostics (7/47), with this final point being mentioned particularly frequently by interviewees in the UK.

Table 10 shows there was little consensus on benefits for the least favoured option, ‘government provides’. However, some interviewees did suggest that having control over the whole diagnostic innovation process, including pricing, could aid cost-effectiveness (10/47). This option was considered more suitable for situations of national crisis (8/47). Some also saw potential benefits if access to testing, suitable evidence and/or test quality improved under this policy (7/47), and this was highlighted most often in the UK. There was more agreement on the pessimistic appraisals. ‘Government provides’ was viewed as potentially very costly and unlikely to be cost effective (19/47), especially by policy makers. There were also concerns from some interviewees in Germany, Spain and the UK, and also particularly from policy makers, that the public sector did not have the capability or capacity to be able to deliver this option (13/47).

## 5.3 Analysis by country and stakeholder groups

The previous section has highlighted the overall results to understand whether and to what extent there is convergence or disagreement between all participants on the favourability of the policy options under appraisal. This section reports the breakdown by country and stakeholder groups, to identify any notable differences in appraisal. These provide additional information about context because policies need to take into account variation, conditionalities and uncertainties linked to specific settings.

Starting with the disaggregated country subsets, Fig. 6 shows that there is no consensus across subgroups as to the preferred policy option, as none share the same ordering. The country subsets reveal that broadly speaking, ‘incentivise use’ features at the top of most country subsets’ merit orders, with the exception of the Greek subset. According to the merit order, collectively Greek interviewees in this exercise ranked ‘protected markets’ highest. Their qualitative comments highlighted that it was seen as being a cost effective option, compared to ‘incentivise use,’ which was seen as difficult to implement due to the need for coordination between stakeholders at different levels (see Table 5). ‘Make pathways’ and ‘Fund R&D’ also featured in the top three preferred options across all country subgroups, and therefore may be applicable across contexts. Finally, ‘protected markets’ and ‘government provides’ do not feature in a consistent manner across country subsets. For instance, a higher proportion of interviewees from Greece and the Netherlands ranked ‘protected markets’ more favourably compared to those in other subgroups. While interviewees from most countries are particularly pessimistic about ‘government provides’, a higher proportion of interviewees from the UK and Spain seem to have a more accepting view of these options.

The merit order for the Greek subset is particularly notable for its differences with other country subgroups. Here, ‘protected markets’ is unusually favoured (recall in Table 5 that 3/7 Greek interviewees saw this as an affordable option), and ‘incentivise use’ is relatively disfavoured, (recall that 3/7 Greek interviewees envisaged the coordination of stakeholders at different levels as a difficulty – see Table 5). Furthermore, options are less distinctly separated in the merit order for Greece than in other countries. A potential explanation for both of these observations centres on the high level of political uncertainty that provided the setting for Greece at the time of the interviews. The Greek government's capacity for AMR policy making was severely constrained due to externally imposed budget controls by the Troika (the International Monetary Fund (IMF), EU and the World Bank) (Mavroudeas, 2017; Mavridis, 2018). As a result Greek health expenditure, which had been higher than average for the EU, was cut by 32% following the Troika's intervention (OECD, 2016a) . In this context, the upholding of an established international intellectual property regime supporting ‘protected markets’ for innovative diagnostics was relatively inexpensive. By contrast, policies dependent on new national expenditure such as ‘fund R&D’ or ‘incentivise use’ were seen as less fitting. One interviewee said “we don't have the money to do R&D here in Greece” and another said that if the government “take a measure which is not very good for the IMF, the IMF pressure them to take it back”.

A higher proportion of interviewees in the Netherlands subset also provided a relatively high ranking for ‘protected markets’ compared to other subgroups, as it was seen as necessary to make the diagnostic market profitable. At the same time, due to effective practice seemingly already in place (recall the Netherlands has low levels of AMR – see Table 3) initiatives such as ‘make pathways’ were seen as less relevant than elsewhere, and most of the interviewees from the Netherlands suggested that the required coordination across the system for this option would be complex and difficult (see Table 7). Indeed, this is a point also often raised by interviewees in Italy, Spain and Greece for ‘make pathways’.

Fig. 7 displays the disaggregated merit orders to explore the variation between stakeholder subsets. Once again it is observed that no two groups share the same merit order, nor does any perspective's ordering entirely agree with the general merit order obtained from aggregation of all interviewee data.

The top-three options generally favoured in the overall aggregation were less commonly placed together in the stakeholder perspective analysis, thus indicating that stakeholder differences may be relevant to consider when forming policies. Only in two groups (policy makers and primary care clinicians) did ‘incentivise use’, ‘fund R&D’ and ‘make pathways’ form the more favoured top three options. There was less consensus over the most favoured option, with only 2/7 stakeholder subsets clearly expecting ‘incentivise use’ to perform best, although the option was rated in the top 3 for the remaining 4/7 stakeholder subsets. Support was notably stronger from interviewees in the diagnostics industry, a high proportion of whom favoured ‘incentivise use’ much more than all other options (recall in Table 5, 3/7 industry interviewees highlighted this could increase revenues and encourage diagnostic development). Pharmacists also often identified ‘incentivise use’ as the option they expected to perform best, for similar reasons to industry representatives. Interviewees in the clinical lab scientists and HTA subsets favoured both ‘fund R&D’ and ‘incentivise use’ (the difference in ratings between the two policy options does not enable a strict conclusion). The merit orders indicate a high proportion of interviewees in the policy maker and primary care clinician subsets expect that both ‘incentivise use’ and ‘make pathways’ would perform best. Secondary care clinicians were the least favourably inclined towards ‘incentivise use’ although it still appears in their top three options.

The evaluation of ‘protected markets’ varied markedly across stakeholder subsets. There was more agreement on the relatively low expectations for ‘enhance revenues’, ranked fifth or below by most groups (4/7) except for more favourable rankings by industry representatives and policy makers (who placed it third and fourth respectively). ‘Government provides’ was often appraised among the least favourable options (4/7), although HTAs favoured it more highly, placing it in third place well above ‘enhance revenues’.

Overall, the comparison of merit orders across perspectives has revealed that the aggregated merit order hides some notable differences in perspective between stakeholder and country interviewee subsets. However, the results also reinforce the aggregated merit order in some respects too. It is particularly clear that from most country and most stakeholder subsets’ perspectives, ‘incentivise use’ is expected to perform well. This is notable because this policy option is not the most prominent in the review of policy literature (see Annex A). Meanwhile ‘enhance revenues’ which enjoys more prominent support in the policy literature does not perform particularly well in most country subsets and the only stakeholder subsets particularly favouring this are policy makers and industry. Less surprisingly, an option that is perhaps more radical, and less discussed in the policy literature, ‘government provides’, is the most widely expected to perform poorly.

## 5.4 Policy mixes

After appraising each option in turn, interviewees were invited to discuss how they would configure an instrument mix from the options that they had appraised. A majority of interviewees (35/47) provided views on possible policy mixes. Table 11 records the number of interviewees identifying conflicts or tensions (shown in the lower triangle) or complementarities and synergies (shown in the upper triangle) for all combinations of policy instrument pairs.

**Table 11:** Complementarity and conflict between policy options as expressed by 35/47 interviewees.

Table 11 shows that those options most favoured in the merit order tend also to be those viewed as being most strongly complementary with each other in a prospective policy mix. Furthermore ‘incentivise use’, the most favoured option in the merit order, is also viewed as the most complementary option overall and identified as most complementary to ‘make pathways’ and ‘fund R&D’ which together make up the other options in the top three favoured policies from the merit order. The interviewee quotes below illustrate some of the reasoning accompanying suggestions these were complementary options. The quotes demonstrate that interviewees recognised these three policies could have an impact in different parts of the innovation system, and why they thought these policies would work better in combination than individually.

“I think ‘making pathways’, ‘incentivise use’, and ‘fund R&D’ [are complementary] … because I think with the pathways you identify the gaps, you can brainstorm about possible solutions, by ‘funding R&D’ you get to those solutions, and with ‘incentivise use’, you get better implementation.” - Secondary care clinician, the Netherlands.

“It's a combination, I wouldn't stick with one policy. It's got to be about ‘incentivise use’ and the patient pathways, if you like if you have NICE [the UK's main health technology assessment agency] guidance with teeth, that would be a similar thing, so I think those two combined, so you've got to have a new policy or a new pathway and then it's got to be adopted because my worry is that we have policies that say let's do this and then no-one does it. There's the execution phase. I think you'd have to combine two. I think ‘make pathways’, I think they already exist, the ‘funding R&D’ is a great thing to do where there's a gap if the commercial market sees no value in developing it, that's where that makes perfect sense.” - Industry executive, the UK.

“In simple words ‘incentivise use’ and ‘enhance revenues’, these are policy options that define the aim and create the motivation, and ‘funding R&D’ and ‘making pathways’ gives the companies tools or support to reach the aim so you should, or one should, combine either of these two (‘incentivise use’ and ‘enhance revenues’) with either of these two (‘fund R&D’ and ‘make pathways’).” - HTA executive, Germany.

“’Fund R&D’ is necessary but not sufficient so there needs to be some money in the short term going into that space so you can kick start the pipeline, that you can encourage some immediate off the mark investment in these new technologies. Beyond that I think focusing on ‘incentivisation of use’, on mechanisms to disrupt the system and to directly encourage and ‘incentivise the use’ of these products is the main way to do it” - Policy maker, UK.

Less than a quarter of those providing views on the policy mix (8/35) identified policies as being in conflicts or tension with each other. Almost all of these tensions were associated with ‘protected markets’ which was identified as being in tension with ‘enhance revenues’, ‘government provides’ or ‘incentivise use’.

## 6 **Discussion and conclusion**

The rise of infectious diseases resistant to available medicines constitutes a major global challenge. This study provides detailed analysis of stakeholders’ views with the aim of informing the development of policy mixes to stimulate diagnostic innovation in order to address AMR. In line with previous research, this study has sought to systematically appraise policy instrument mixes as seen under a diverse range of specialist stakeholder perspectives. Particular attention has been paid here to the conditionalities attending the fit between particular policy options and specific national settings as well as to uncertainties within, and ambiguities across, perspectives and to issues bearing on the design of policy mixes comprising a diversity of individual instruments (Borrás and Edquist, 2013).

### 6.1 Contribution to general prospective appraisal of innovation instrument mixes

The study has demonstrated a newly enhanced form of MCM in order to capture quantitative and qualitative appraisal by all interviewees, which could be applied to support systematic appraisals in innovation policy. By focusing on systematic and comprehensive pairwise analysis of policy options, it has been possible to combine representation of ambiguities in stakeholders’ relative degree of pessimism and optimism for each option in appraisal, together with the uncertainties they perceive about the future performance of individual options. This novel method builds on different traditions in decision analysis in order to illuminate key factors underpinning stakeholders’ inclinations to favour some options over others when considering a portfolio of options. Without disregarding crucial aspects of variance and uncertainty, the method helps to inform policy making by deriving a picture of overall merit orders.

Although this article is primarily focused on the design of policy making concerning diagnostic innovation to address AMR, the same broad approach might reasonably be expected to hold relevance for wider applications in policy appraisal, innovation foresight, technology assessment and research evaluation. Whilst individual policy instruments are often subject to evaluation, this is rarely as balanced across diverse options and perspectives, nor as attentive as here, to uncertainties and ambiguities, nor (crucially) as explicit in the consideration of the design of instrument mixes. The hybrid approach described here may help facilitate wider efforts to integrate the contrasting qualities of quantitative and qualitative approaches.

### 6.2 **The design of policy instrument mixes to stimulate diagnostic innovation against AMR**

#### 6.2.1 Findings on ‘common ground’ and differences

The emerging threat to global health from AMR has motivated an international response with a surge of policy reports and research studies published in recent years. These express a widespread view that systemic responses to AMR are required by national innovation systems, including specific support for diagnostic innovation. These documents have variously advocated a range of different policy options, although not always with rigorous explanations of how the favoured options were identified or on what grounds. Often neglected is how policy needs vary between countries and change over time.

Focusing on six European countries, this study provides a systematic, rigorous and transparent approach to appraising a wide range of policy options, including those most prominent in the extant literature, as well as some less frequently mentioned. Among the many conditionalities this analysis permits to be interrogated, are the particular circumstances of each of the six different countries addressed. It is in respect of associated variations, for instance in the maturity of the diagnostics innovation system or the extent of the national AMR threat, that analytical disaggregation may be necessary to reveal the fit of particular options with anticipated needs in a given setting. Also highly relevant for the design of options, are the contrasting views on favoured policy options displayed under different stakeholder perspectives.

Yet despite the care taken to elicit and assimilate variations in appraisals from diverse positions, a key feature that emerges in this analysis is a relative consistency in respect of the options revealed to be most and least favoured. That such a finding is resolvable (in a suitably qualified way), is all the more salient for being the result of an approach that is so dedicated to illuminating diversity in so many different ways.

Those features most broadly agreed in these merit orderings suggests strongest interviewee support for the option to ‘incentivise use’ of clinical diagnostic tests, with weakest interviewee support for the option where ‘government provides’ diagnostic innovations directly without reliance on commercial actors. An overall tendency to most favour ‘incentivise use’ was evident across subsets of interviewees for five of the six countries studied (the exception being Greece). For interviewee subsets from Germany, Spain, the UK and Italy, common ground also emerged around relatively favourable appraisals of ‘fund R&D’ and ‘make pathways’ with these joining ‘incentivise use’ as the top three most consistently favoured policies. In these same four interviewee subsets, ‘enhance revenues’ and ‘protected markets’ join ‘government provides’ as the three options that tend to be least favoured overall.

However, this broad-brush picture is under-laid by a number of key contextual qualifications. That there is evidently no one-size-fits-all solution, makes these nuances especially important in determining the appropriate mixes for particular national settings. For instance, this point was particularly clear from the results obtained from Dutch and Greek interviewees. That the ‘make pathways’ option was relatively less strongly favoured in comparative terms in the Dutch subset, related to the comparatively well-supported healthcare system and relatively low incidence of AMR in this country. For a higher proportion of the Greek interviewees, by contrast, there was less confidence in implementing policies requiring new national expenditure (in a country still recovering from the financial crisis at the time interview data was collected (Hardouvelis et al., 2018; Mavridis, 2018)). However, there was comparatively greater confidence in the Greek conext in policy options rooted in external frameworks, such as the EU intellectual property regime that is central to providing ‘protected markets’ for innovative products.

Also important in these relatively broad terms is the result that the three policy options identified above as more highly favoured across diverse stakeholders’ views on an individual basis, also tended to come most positively to the fore in interviewees’ appraisals of their potential to be complementary with each other (rather than in tension) when included together as parallel policy instruments in the same mix. Despite the important diversity across different settings and perspectives, a consistent picture across a high proportion of the interviewees from Germany, Italy, Spain and the UK was to favour a combination of ‘incentivise use’ with ‘fund R&D’, as well as ‘incentivise use’ and ‘make pathways’. It is notable that the counterparts to ‘incentives use’ in each respect (‘fund R&D’ and ‘make pathways’) also independently featured as the next most compatible pairing for mixes. The findings on interviewees’ views regarding instrument mixes are all the more persuasive for being based on data collected at the close of the interviews, outside of the MCM process used to appraise individual instruments.

#### 6.2.2 Differences between stakeholder perspectives

It is a particular facility of MCM that disaggregated pictures can easily be derived for contrastingly-defined groupings of perspectives. Distinguishing for this purpose between appraisals undertaken by interviewees in subsets identified as industry executives, pharmacists, primary care clinicians, and policy makers, a picture emerges, that the ‘incentivise use’ option tended to be highly favoured, with the ‘make pathways’ option ranking second. Clinical lab scientists and HTA experts, by contrast, tended to appraise ‘fund R&D’ most favourably, closely followed by ‘incentivise use’. Secondary care clinicians tended to be more favourable towards ‘fund R&D’, closely followed by ‘protected markets’.

Lower down the merit orders there were more marked variations between groups. This is perhaps not unexpected given the different interests in play – with different parts of the innovation system being better known to some stakeholder groups than others and different policy instruments bringing resources to different parts of the innovation system. These differences accepted, it tended notably to be ‘incentivise use’, ‘fund R&D’ and ‘make pathways’ that featured most frequently as the highest ranked options across all these stakeholder groups. This tendency towards common ground across such diverse perspectives arguably diminishes the queries that might otherwise very reasonably be raised about how sensitive these findings might be, to issues of perceived ‘representativeness’ with respect to variously-definable contrasting perspectives in appraisal. Analysis of disaggregated perspectives in MCM does not escape the kinds of question that are always applicable with any kind of analysis of categorised social groupings (O'Neill, 2001). But where patterns across contrasting possible groupings display strong commonalities, then conclusions may be correspondingly more confident.

#### 6.2.3 Illumination of qualitative rationales and their application

A core research question in this study, asked *why* it is that different options are variously favoured or not. Issues in this regard that came most prominently to the fore include perceptions of relative cost-effectiveness, or the challenging nature of effective co-ordination across complex innovation systems. The desirability for co-operation across public and private sectors was often raised, and there were also concerns that policies such as ‘protected markets’ and ‘enhance revenues’ should not overly reward the private sector, particularly for products that might not sufficiently help to manage AMR.

While it is arguably only appropriate to highlight here the most prominent themes (mentioned above) we note that the full list of reasons given to support or oppose particular options reveals both more common and rare, but still potentially important, caveats relating to the configuration of policies for particular situations. The extensive contextual information revealed by this study may be of value to policy makers concerned with the current and future contexts for the policy instruments they are seeking to establish. With this in mind, Annex D of the supplementary materials provides a checklist of prompts (based on all relevant points raised by interviewees) for policy makers engaged in the design of particular instruments.

#### 6.2.4 Relevance to the existing AMR policy literature

The role of diagnostics in addressing the challenge of AMR is often overlooked (Ghani et al., 2015) or else held subsidiary to the goal of bringing new antibiotics into use (e.g. O'Neill, 2016). This study has demonstrated how policy could productively encourage innovation in diagnostic testing in order to better manage AMR. The study summarises the main types of policy options to stimulate diagnostic innovation, from a broad extant literature, and systematically appraises these through interviews with relevant experts. Naturally the resulting evidence supports the development of combinations of policies suitable for specific contexts. Factors that influence the performance of different policies are identified, which could enhance implementation and outcomes. While a uniform prescription is not supported, it is notable that the policy option that emerged in this process as tending to be more favoured, ‘incentivise use’, was relatively lacking in prominence in the extant policy literature. This family of instruments was only 6th out of 8, based on the literature-based tally summarised in Table 1 with policy options to ‘make pathways’, ‘enhance revenues’, and ‘fund R&D’ being much more frequently mentioned. It is therefore all the more notable that a wide range of stakeholders from across a diversity of countries should broadly tend relatively to disfavour a number of policy options that are prominent in the literature, by comparison with a particular policy option (‘incentivise use’) that is conventionally comparatively less attended to. Indeed, even in the industry subset (which might be thought the key beneficiary of those policies most emphasised in the extant policy literature), a high proportion of interviewees also tended quite strongly to favour ‘incentivise use’ over all the other appraised options.

#### 6.2.5 Limitations of this study

As in any analysis of a complex, dynamic, uncertain and diverse field, this study displays a number of limitations associated with methodological design and implementation. One of these is the necessarily compressed account forced by the journal article format on the discussion that is possible on methodological design and the nuances of findings. Many features of the MCM process are necessarily side-lined here in order to set out the main focus of analysis. These are however published in the MCM manual (Coburn et al., 2019).

This said, one limitation that is often perceivable in ‘thick’ interpretive or hybrid quantitative / qualitative research like this is that, by comparison with conventional statistical analysis, the number of interviewees (47) is relatively small. Perhaps most pertinent here, is that the assumptions and norms of mainstream statistical analysis (themselves under increasing question nowadays (Ioannidis, 2005)(Cumming, 2014)(Mayo, 2018)) – like high ‘n’ and statistical representativeness – are not directly applicable to incommensurable methodologies like Q method or MCM (Stephenson, 1953; McKeown and Thomas, 1988; Coburn et al., 2019). More relevant here are qualities discussed earlier (Section 4.2) relating to ‘non-probabilistic’ (Parker et al., 1998) ‘case study logic’ rather than ‘sampling logic’ (Yin, 2009) – of ‘validity’ (Mitchell, 1983), ‘inclusiveness’ (O'Neill, 2001), ‘legitimacy’ (Chilvers, 2009) and ‘transparency’ (Stirling, 2003) more than ‘statistical representativeness’ (Smith and Wales, 2000).

It is important in MCM as in other in-depth methods to qualify that the merit orders discussed above for specific country perspectives, may each be strongly influenced by the conditions of elicitation. And it is here that correspondence reported between the merit orders derived across all 47 interviews and those associated with specific perspectives within this, are suggestive of a reassuring degree of robustness for the findings that are the main emphasis in this article. However, as the analysis also shows, this overall merit order is not fully aligned with any particular subgroup of interviewees based on either country or stakeholder perspective, so corresponding caution is required in applying overall results to any particular setting.

It is intrinsic to this field, that a second legitimately perceivable limitation is, in some ways, of the opposite kind: that it can always be judged that some relevant stakeholder perspective has been excluded. For example, focusing as this study does on specialist understandings, both the ‘patient voice’ and the perspectives of distinguishable ‘publics’ are notably absent here. Of course, that such questions can be raised at all, reflects the relatively unusual transparency here (for policy appraisal in general), with regard to the inclusion of constituencies. And in this case, this exclusion reflects the lack of a strong patient or public voice in the national debates and committees around AMR in some countries (whatever this may imply).

Thirdly, care was taken at interview to ensure interviewees all received the same introductory materials and facilitation by interviewers. Nonetheless it is always the case in any method, that it cannot be excluded that detailed tacit interpretations adopted by individual participants in different settings may attach contrasting detailed associations to terms used to describe the policy instruments. The care taken in MCM to document qualitative responses provides a check on this risk, but does not eliminate it.

A fourth limitation arises from MCM interviews being highly systematically structured and taxing on individual deliberation by interviewees, as well as being demanding on interviewers. It is as a result of this feature – and the principle that results will not be used in MCM unless the interviewee is fully confident that they adequately reflect their personal view – that data obtained in this study could not be used from three interviews (out of 50) because of implementation difficulties under the circumstances or in the time available.

A fifth major limitation, of course, is the restrictive focus just on Western European states. This is all the more salient, because (despite some regional variations and global gaps in the available data (WHO, 2017)), these have lower rates of AMR and more highly resourced healthcare services than many countries outside Europe (Collignon et al., 2018). The obvious remedy in this respect, is to complement this study with other work of broader reach.

#### 6.2.6 Further research

The magnitude of the threat posed by AMR and the complexity of the required response strategies provide ample incentives for further studies on innovation policy in relation to the detection and treatment of drug resistant pathogens. For researchers also seeking to focus on the role of diagnostics, it may be informative to build on this study by consulting a wider range of individuals to confirm these results in particular national settings. Looking beyond the European countries studied here, similar studies may be needed for other countries, particularly in resource poor settings where AMR is most prevalent and deadly and where perhaps the policy instruments required differ from those favoured in the large European economies predominant in the sample studied here. It is important to recognise that the management of AMR amounts to a global and intergenerational arms race between humans and microbial pathogens, requiring significant investments in diagnostic innovation. It is therefore appropriate to monitor the mix of policy instruments applied to manage diagnostic innovation focused on AMR, and it may also be relevant to study the sequencing of interventions over the long term (Cunningham et al., 2013).

## Declaration of Competing Interest

The authors declare that they have no known competing financial interests or personal relationships that could have appeared to influence the work reported in this paper.

## Appendix Supplementary materials

Image, application 1

## Footnotes

1 ‘All you need to know about the antibiotic apocalypse’, BBC News website, 10^th^ October (Nogrady, 2016); ‘Antibiotic Apocalypse: Doctors sound warning over drug resistance’, The Observer, 8^th^ October (McKie, 2017).
