# Supplementary material for: Appraising research policy instrument mixes: a multicriteria mapping study in six European countries of diagnostic innovation to manage antimicrobial resistance
Source: Res Policy. 2021 May;50(4):104140. doi: 10.1016/j.respol.2020.104140 (PMC8039188; doi:10.1016/j.respol.2020.104140)
Supplement: Supplementary file 1 [file mmc1.docx]

**Annex A. Selection of policy options for MCM interviews**

Annex A describes how the policy options to encourage diagnostic innovation to address AMR were selected for discussion with stakeholders. MCM relies on all interviewees appraising a common portfolio of options. While the same core options are evaluated by all interviewees, they are free to choose any criteria they deem appropriate to evaluate the options, as long as all options are evaluated by the same criteria by a particular interviewee. The MCM process thus involves appraising every option in the portfolio against the same self-specified set of criteria and this cyclical process is time consuming and cognitively demanding. It is therefore important to limit the number of options discussed at interview, with around six being optimal (Coburn & Stirling, 2016). To make best use of this relatively small number of options, it is helpful to choose a set of ‘core’ options that are collectively diverse and clearly distinguished from each other with respect to certain characteristics that are pertinent to the field in question (Coburn & Stirling, 2016). Interviewees were also invited add their own additional options if they thought that it was important to supplement the core options provided (see Annex C).

Policy options selected for discussion at interview were derived from those already in circulation in the policy literature. A systematic search was undertaken with the initial aim of identifying a wide range of policy types focusing on a particular policy goal: The development and use of diagnostic tests to manage AMR. Policy reports and academic articles which discuss diagnostic innovation and AMR were gathered by performing a systematic search for documents authored since 2000, using the following search terms: ‘antimicrobial resistance diagnostics’, ‘antibiotic resistance diagnostics’, and ‘AMR diagnostics’. Additional AMR strategy documents from prominent bodies developing policy options (e.g. WHO, OECD, and EU) were added to the resulting list. A set of 54 relevant source documents were collected, with the vast majority published within the last seven years (28 were published in the years 2015-2016, a further 20 between 2010-2014, while just five pre-date 2010).

Sources were read to identify policy options with the resulting options initially classified into ten categories, which themselves were classified as Science-Push models (three categories), Market-Pull models (seven categories) and an additional ‘other’ category (see Table 1). In a second phase, options were grouped by the mechanism that they targeted. In this way, the following categories were combined: guaranteed profitable prices for tests, prize competitions, and strengthened intellectual property (IP) rights – these all focus on incentivising industry diagnostics developers by enhancing the revenues associated with diagnostic tests that reach the market, and so these were grouped together under the umbrella term ‘enhance revenues’. Similarly, improving the infrastructure for R&D and improving the infrastructure for commercialisation both make the pathways from research through to commercialisation easier to navigate for test developers and so these were grouped together as ‘make pathways’. Finally, providing incentives to encourage healthcare professionals to use diagnostic tests, removing disincentives, and ensuring that funding for antibiotics and for tests is not siloed are all ways of incentivising the use of tests and these were grouped together in a category called ‘incentivise use’. After merging the options (see Figure 1), six categories of instruments were selected based on the distinctiveness of the options chosen. It is notable that this did not result in all of the more commonly occurring options from the literature being selected for discussion at interview. Two sets of options were frequently mentioned in the literature but not selected. The first was ‘communication and dissemination’, which was seen as an essential part of the implementation of other policies, and also not distinct enough to warrant specific focus. Secondly, those policies focused on actions above the nation level were excluded, given the focus of the study on national systems of innovation. Additionally, two categories that were rarely mentioned in the literature were included, so as to provide an opportunity to discuss the status quo (described as firms competing for market share in the context of IP rights to protect their R&D investments), and an option based on the assumption that open science or public provision was needed, as opposed to private/ commercial diagnostic innovation. These options were renamed to provide short, easily recognisable names, suitable for use in interviews. The traditional platform model was renamed ‘protected markets’, while the non-commercial, open science model was renamed ‘government provides’.

***Figure 1: Policy options selected for discussion***

***Table 1: Long list of policy options by category and source***

|  |  | **Instruments** | | | | | | | | | | |
| --- | --- | --- | --- | --- | --- | --- | --- | --- | --- | --- | --- | --- |
|  |  | **Push models** | | | **Pull models** | | | | | | | **Other** |
| **Type of publication** | **Citation** | **R&D grant funding** | **Infrastructure for R&D** | **Infrastructure for commercialisation** | **The traditional platform model** | **Guaranteed profitable prices** | **Prize competitions** | **Encourage test use** | **Communication & dissemination** | **Strengthened intellectual property rights** | **‘Open science’** | **Global / overarching / other issues** |
| **AMR diagnostics reports** | (O’Neill, 2015) | y | y | Y | y | y | y | Y | y | y |  | y |
|  | (Morel et al., 2016) | y | y |  | y |  | y | y |  |  |  |  |
|  | (Plüddemann et al., 2015) |  | y | Y |  |  |  | y | y |  |  | y |
|  | (Peeling & Boeras, 2016) |  | y | Y |  |  |  |  | y |  |  | y |
| **AMR reports** | (O’Neill, 2016) | y |  |  |  | y |  | y |  |  |  | y |
|  | (Merrett, 2013) | y | y | Y |  | y |  | y | y | y |  | y |
|  | (EHP Committee on AMR, 2016) | y |  |  |  | y |  |  |  |  |  |  |
|  | (IFPMA, 2016) | y | y | y |  | y |  | y |  |  |  | y |
|  | (Plahte & Rottingen, 2015) | y | y |  |  | y |  |  |  | y |  |  |
|  | (Renwick, Brogan, & Mossialos, 2014) | y | y | y |  | y | y |  | y | y |  | y |
|  | (WHO, 2015) | y |  |  |  |  |  |  | y |  |  | y |
|  | (Sharma & Towse, 2011) | y | y | y |  | y | y | y |  | y |  | y |
|  | (BEAM Alliance, 2015) | y |  | y |  | y |  |  |  |  |  |  |
|  | (Nugent, Back, & Beith, 2010) | y | y | y |  | y |  | y |  |  |  | y |
| **Diagnostics reports** | (Deloitte Centre for Health Solutions, 2013) |  | y | y |  | y |  | y | y |  |  | y |
| **AMR strategy reports** | (DH & DEFRA, 2013) | y |  |  |  |  |  | y | y |  |  | y |
|  | (WHO, 2012) | y | y | y |  | y | y | y | y | y |  | y |
|  | (The White House, 2015) | y | y | y |  | y | y | y |  |  |  |  |
|  | (Cecchini, Langer, & Slawomirski, 2015) | y | y | y |  | y | y |  | y | y | y | y |
|  | (AEMPS, 2015) |  | y |  |  |  |  | y | y |  |  |  |
|  | (Ministère chargé de la Santé, 2016) | y | y | y |  |  |  |  | y |  |  |  |
|  | (Federal Ministry of Health, 2008) | y |  |  |  | y |  | y | y |  |  |  |
| **Antibiotic business models reports** | (Jaczynska, Outterson, & Mestre-Ferrandiz, 2015) | y | y | y |  | y |  | y |  |  |  | y |
|  | (Clift et al., 2015) | y |  |  |  | y |  |  |  | y |  | y |
|  | (Outterson, 2014) | y |  |  |  | y | y | y |  | y |  |  |
|  | (Zorzet & So, 2014) |  | y | y |  |  |  |  |  |  |  |  |
|  | (Kieny, 2015) | y |  |  |  | y | y |  |  |  |  | y |
|  | (Renwick, Brogan, & Mossialos, 2016) | y | y | y |  | y |  | y |  | y |  | y |
|  | (Renwick, Simpkin, & Mossialos, 2016) | y | y | y |  |  |  |  |  |  |  | y |
| **Reports from workshops / meetings** | (MRC & JPIAR, 2015) |  | y | y |  |  |  |  | y |  |  | y |
|  | (CDC & AdvaMedDx, 2016) |  | y |  |  |  |  | y | y |  | y | y |
|  | (Jones et al., 2016) |  |  | y |  |  |  | y | y |  |  |  |
|  | (Wellcome Trust, 2015) | y | y | y |  |  |  | y | y |  |  |  |
|  | (Cecchini, 2016) |  |  |  |  |  |  |  | y |  |  |  |
|  | (Chatham House, 2013) | y |  | y |  | y | y | y | y | y |  | y |
| **AMR diagnostics journal articles** | (Towse & Garrison, 2013) |  |  | y |  | y |  |  |  | y |  |  |
|  | (Garau, Towse, Garrison, Housman, & Ossa, 2013) |  |  |  |  | y |  |  |  |  |  |  |
|  | (Garrison & Towse, 2014) |  |  |  |  | y |  |  |  |  |  |  |
|  | (Hopkins & Hogarth, 2012) |  |  |  | y |  |  |  |  | y | y |  |
|  | (Peterson & Dalhoff, 2004) |  |  | y |  |  |  |  |  |  |  |  |
|  | (Finch, 2007) | y |  | y |  |  |  |  |  |  |  | y |
|  | (Okeke et al., 2011) | y | y | y |  | y |  | y | y |  |  |  |
|  | (Rice, 2011) |  |  |  |  |  |  | y | y |  |  |  |
| **AMR journal articles** | (Spellberg et al., 2011) | y | y | y |  | y |  |  | y | y |  | y |
|  | (Laxminarayan et al., 2013) |  |  | y |  |  |  |  | y |  |  | y |
|  | (Brogan & Mossialos, 2016) | y |  | y |  | y |  |  |  |  |  | y |
|  | (Harbarth & Samore, 2005) |  |  | y |  | y |  | y | y |  |  |  |
|  | (Leung, Weil, & Nakatani, 2011) | y |  | y |  |  |  | y | y |  |  |  |
|  | (So, Ruiz-Esparza, Gupta, & Cars, 2012) | y | y |  |  | y |  |  |  |  |  | y |
|  | (Laxminarayan, Sridhar, Blaser, Wang, & Woolhouse, 2016) | y |  |  |  | y |  |  | y |  |  | y |
|  | (Spellberg et al., 2008) | y |  | y |  | y |  |  | y | y |  |  |
| **Antibiotic business models journal articles** | (Rex & Outterson, 2016) |  |  |  |  | y |  |  |  |  |  | y |
|  | (Outterson et al., 2016) |  |  |  |  | y |  |  |  | y |  |  |
|  | (Outterson, Powers, Daniel, & Mcclellan, 2015) | y |  |  |  | y |  |  |  |  |  | y |

**Bibliography for Annex A**

AEMPS. (2015). *Strategic Action Plan to reduce the risk of selection and dissemination of antibiotic resistance*. Madrid: Spanish Agency of Medicines and Medical Devices (AEMPS).

BEAM Alliance. (2015). BEAM Alliance Position Paper: Key Actions to Reinvigorate Investment and R & D in the antibacterial field Now. Paris: The BEAM Alliance. <https://doi.org/10.1377/hlthaff.2014.1003.1>

Brogan, D. M., & Mossialos, E. (2016). A critical analysis of the review on antimicrobial resistance report and the infectious disease financing facility. *Globalization and Health*, *12*(8). <https://doi.org/10.1186/s12992-016-0147-y>

CDC, & AdvaMedDx. (2016). *A Public Health Perspective on Antimicrobial Resistance Diagnostics: Meeting Summary and Opportunities to Address Challenges*. Atlanta: Centers for Disease Control and Prevention and AdvaMedDx.

Cecchini, M. (2016). *Promoting Rational Use of Antimicrobials: Expert Meeting: Releasing Health System Resources for Better Value Care: Tackling Ineffective Health Spending and Waste*. Paris: Directorate for Employment, Labour and Social Affairs, Health Committee, OECD.

Cecchini, M., Langer, J., & Slawomirski, L. (2015). *Antimicrobial Resistance in G7 Countries and Beyond: Economic Issues, Policies and Options for Action*. Paris: OECD.

Chatham House. (2013). *Antimicrobial Resistance: Incentivizing Change towards a Global Solution: Global Health Security Summary*. London: Chatham House.

Clift, C., Gopinathan, U., Morel, C., Outterson, K., Røttingen, J.-A., & So, A. (2015). *Towards a New Global Business Model for Antibiotics: Delinking Revenues from Sales: Executive Summary*. London: Chatham House. Retrieved from <https://www.chathamhouse.org/publication/towards-new-global-business-model-antibiotics-delinking-revenues-sales>

Coburn, J., & Stirling, A. (2016). *Multicriteria Mapping Manual, Version 2.0, SWPS 2016-21*. Brighton: SPRU - Science Policy Research Unit, University of Sussex.

Deloitte Centre for Health Solutions. (2013). *Working differently to provide early diagnosis: Improving access to diagnostics*. London: Deloitte LLP.

DH, & DEFRA. (2013). *UK Five Year Antimicrobial Resistance Strategy 2013 to 2018*. London: Department of Health, Department for Environment Food and Rural Affairs. Retrieved from <https://www.gov.uk/government/uploads/system/uploads/attachment_data/file/244058/20130902_UK_5_year_AMR_strategy.pdf>

EHP Committee on AMR. (2016). *Tackling a Global Public Health Crisis*. Brussels: European Health Parliament Committee on Antimicrobial Resistance.

Federal Ministry of Health. (2008). *DART: German Antimicrobial Resistance Strategy*. Berlin: Federal Ministry of Health.

Finch, R. (2007). Innovation — drugs and diagnostics. *Journal of Antimicrobial Chemotherapy*, *60*(Suppl i), i79–i82. <https://doi.org/10.1093/jac/dkm165>

Garau, M., Towse, A., Garrison, L., Housman, L., & Ossa, D. (2013). Can and should value-based pricing be applied to molecular diagnostics? *Personalized Medicine*, *10*(1), 61–72. <https://doi.org/10.2217/pme.12.99>

Garrison, L. P., & Towse, A. (2014). Personalized Medicine: Pricing and Reimbursement Policies as a Potential Barrier to Development and Adoption, Economics of. In A. J. Culyer (Ed.), *Encyclopedia of Health Economics* (Vol. 2, pp. 484–490). San Diego: Elsevier. <https://doi.org/10.1016/B978-0-12-375678-7.01210-4>

Harbarth, S., & Samore, M. H. (2005). Perspective: Antimicrobial Resistance Determinants and Future Control. *Emerging Infectious Diseases*, *11*(6), 794–801.

Hopkins, M. M., & Hogarth, S. (2012). Biomarker patents for diagnostics: problem or solution? *Nature Biotechnology*, *30*(6), 498–500. <https://doi.org/10.1038/nbt.2257>

IFPMA. (2016). *Declaration by the Pharmaceutical, Biotechnology and Diagnostics Industries on Combating Antimicrobial Resistance*. Geneva: International Federation of Pharmaceutical Manufacturers & Associations.

Jaczynska, E., Outterson, K., & Mestre-Ferrandiz, J. (2015). *Business Model Options for Antibiotics: Learning from Other Industries*. London: Chatham House & Big Innovation Centre.

Jones, I., Diver, M., Gertler, N., Rex, J., Spencer, K., Jinks, T., & Seabrook, R. (2016). Four diagnostic strategies for better-targeted antibiotic use. London: Wellcome Trust.

Kieny, M.-P. (2015). Creating an Intergovernmental Consortium for New Antibiotics: A New Development Model. In J. Carlet (Ed.), *AMR Control* (pp. 26–32). Woodbridge: Global Health Dynamics.

Laxminarayan, R., Duse, A., Wattal, C., Zaidi, A. K. M., Wertheim, H. F. L., Sumpradit, N., … Cars, O. (2013). The Lancet Infectious Diseases Commission: Antibiotic resistance — the need for global solutions. *The Lancet Infectious Diseases*, *13*(12), 1057–1098. <https://doi.org/10.1016/S1473-3099(13)70318-9>

Laxminarayan, R., Sridhar, D., Blaser, M., Wang, M., & Woolhouse, M. (2016). Achieving global targets for antimicrobial resistance. *Science*, *9286*(August).

Leung, E., Weil, D. E., & Nakatani, H. (2011). Perspectives: The WHO policy package to combat antimicrobial resistance. *Bull World Health Organ*, *89*, 390–392. <https://doi.org/10.2471/BLT.11.088435>

Merrett, G. L. B. (2013). *Chatham House Briefing Paper: Tackling Antibiotic Resistance for Greater Global Health Security*. London: Chatham House.

Ministère chargé de la Santé. (2016). *Plan national d’alerte sur les antibiotiques 2011-2016*. Paris: Ministère chargé de la Santé.

Morel, C., McClure, L., Edwards, S., Goodfellow, V., Sandberg, D., Thomas, J., & Mossialos, E. (2016). *Ensuring innovation in diagnostics for bacterial infection: Implications for policy*. Brussels: European Observatory on Health Systems and Policies, World Health Organization.

MRC, & JPIAR. (2015). *Identifying the pathway to diagnostic development: Report from the workshop on 1 May 2015*. London: Medical Research Council and Joint Programming Initiative on Antimicrobial Resistance.

Nugent, R., Back, E., & Beith, A. (2010). *The Race Against Drug Resistance: A Report of the Center for Global Development’s Drug Resistance Working Group*. Washington, DC: Center for Global Development. Retrieved from <http://cgdev.org/files/1424208_file_DRWG_brief_FINAL.pdf%5Cnpapers2://publication/uuid/ECFF80C7-4401-43D4-A88D-F2315D7BEF78>

O’Neill, J. (2015). *Rapid diagnoistics: Stopping unnecessary use of antibiotics*. London: Review on Antimicrobial Resistance. Retrieved from <http://amr-review.org/sites/default/files/Paper-Rapid-Diagnostics-Stopping-Unnecessary-Prescription-Low-Res.pdf>

O’Neill, J. (2016). Tackling Drug-Resistant Infections Globally: Final Report And Recommendations. London: Review on Antimicrobial Resistance.

Okeke, I. N., Peeling, R. W., Goossens, H., Auckenthaler, R., Olmsted, S. S., Lavison, J.-F. De, … Nordqvist, K. (2011). Diagnostics as essential tools for containing antibacterial resistance. *Drug Resistance Updates*, *14*, 95–106. <https://doi.org/10.1016/j.drup.2011.02.002>

Outterson, K. (2014). *New Business Models for Sustainable Antibiotics*. London: Centre on Global Health Security Working Group Papers, Chatham House.

Outterson, K., Gopinathan, U., Clift, C., So, A. D., Morel, C. M., & Røttingen, J.-A. (2016). Delinking Investment in Antibiotic Research and Development from Sales Revenues: The Challenges of Transforming a Promising Idea into Reality. *PLOS Medicine*, *13*(6), 1–7. <https://doi.org/10.1371/journal.pmed.1002043>

Outterson, K., Powers, J. H., Daniel, G. W., & Mcclellan, M. B. (2015). Repairing The Broken Market For Antibiotic Innovation. *Health Affairs*, *34*(2), 277–285. <https://doi.org/10.1377/hlthaff.2014.1003>

Peeling, R. W., & Boeras, D. I. (2016). Diagnostic Innovation for Antimicrobial Resistance. In J. Carlet (Ed.), *AMR Control* (pp. 40–44). Woodbridge: Global Health Dynamics.

Peterson, L. R., & Dalhoff, A. (2004). Towards targeted prescribing: will the cure for antimicrobial resistance be specific, directed therapy through improved diagnostic testing? *Journal of Antimicrobial Chemotherapy*, *53*(6), 902–905. <https://doi.org/10.1093/jac/dkh187>

Plahte, J., & Rottingen, J.-A. (2015). Antibiotic Innovation– Some Lessons from the WHO Processes on Public Health, Innovation and Intellectual Property. In J. Carlet (Ed.), *AMR Control* (pp. 18–25). Woodbridge: Global Health Dynamics.

Plüddemann, A., Onakpoya, I., Harrison, S., Shinkins, B., Tompson, A., Davis, R., … Heneghan, C. (2015). *Position Paper on Anti-Microbial Resistance Diagnostics*. Oxford: Centre for Evidence-Based Medicine, Nuffield Department of Primary Care Health Sciences, University of Oxford. <https://doi.org/10.13140/RG.2.1.1135.9846>

Renwick, M. J., Brogan, D. M., & Mossialos, E. (2014). *A Critical Assessment of Incentive Strategies for Development of Novel Antibiotics*. London: LSE.

Renwick, M. J., Brogan, D. M., & Mossialos, E. (2016). A systematic review and critical assessment of incentive strategies for discovery and development of novel antibiotics. *The Journal of Antibiotics*, *69*(2), 73–88. <https://doi.org/10.1038/ja.2015.98>

Renwick, M. J., Simpkin, V., & Mossialos, E. (2016). *International and European Initiatives Targeting Innovation in Antibiotic Drug Discovery and Development: The Need for a One Health – One Europe – One World Framework Report* (Vol. 44). London: LSE Health, Department of Social Policy, London School of Economics and Political Science and European Observatory on Health Systems and Policies.

Rex, J. H., & Outterson, K. (2016). Personal View: Antibiotic reimbursement in a model delinked from sales: a benchmark-based worldwide approach. *Lancet Infect Dis*, *16*(April), 500–505.

Rice, L. B. (2011). Rapid Diagnostics and Appropriate Antibiotic Use. *Clinical Infectious Diseases*, *52*(Suppl 4), S357–S360. <https://doi.org/10.1093/cid/cir051>

Sharma, P., & Towse, A. (2011). *New Drugs to Tackle Resistance: Analysis of EU Policy Options*. London: Office of Health Economics.

So, A. D., Ruiz-Esparza, Q., Gupta, N., & Cars, O. (2012). 3Rs for innovating novel antibiotics: sharing resources, risks, and rewards. *BMJ*, *344*(e1782). <https://doi.org/10.1136/bmj.e1782>

Spellberg, B., Blaser, M., Guidos, R. J., Boucher, H. W., Bradley, J. S., Eisenstein, B. I., … Gilbert, D. N. (2011). Combating Antimicrobial Resistance: Policy Recommendations to Save Lives. *Clinical Infectious Diseases*, *52*(Suppl 5), S397–S428. <https://doi.org/10.1093/cid/cir153>

Spellberg, B., Guidos, R., Gilbert, D., Bradley, J., Boucher, H. W., Scheld, W. M., … Diseases, I. (2008). The Epidemic of Antibiotic-Resistant Infections: A Call to Action for the Medical Community from the Infectious Diseases Society of America. *Clinical Infectious Diseases*, *46*, 155–164. <https://doi.org/10.1086/524891>

The White House. (2015). *National Action Plan for Combating Antibiotic-Resistant Bacteria*. Washington: The White House.

Towse, A., & Garrison, L. P. (2013). Economic incentives for evidence generation: Promoting an efficient path to personalized medicine. *Value in Health*, *16*(6 SUPPL.), S39–S43. <https://doi.org/10.1016/j.jval.2013.06.003>

Wellcome Trust. (2015). *A patient centric approach to the integrated use and development of diagnostic tests, diagnostic strategies and their therapeutics for treatment of infection: Workshop Summary*. London: Wellcome Trust.

WHO. (2012). *The evolving threat of antimicrobial resistance: Options for action*. Geneva: World Health Organisation.

WHO. (2015). *Global action plan on antimicrobial resistance*. Geneva: World Health Organisation.

Zorzet, A., & So, A. D. (2014). *WHO Proposal for Health R&D Demonstration Project: Building a Diagnostic Innovation Platform to Address Antibiotic Resistance*. Uppsala and Durham: ReAct – Action on Antibiotic Resistance and Program on Global Health and Technology Access.

**Annex B: Interviewee Briefing Document for Foresight Study on European Stakeholder Appraisal of Diagnostic Tests to Manage Antimicrobial Resistance**

Thank you for agreeing to take part in this ‘Foresight Study on European Stakeholder Appraisal of Diagnostic Tests to Manage Antimicrobial Resistance project’. Before your interview, please read the following information on the research project and what your contribution will involve.

**The interview focus**

We are interviewing key opinion leaders in different European countries to explore new ways of encouraging the development and use of appropriate diagnostic tests (or “diagnostics” for short) to address the AMR challenge. In theory, diagnostics have the potential to rapidly aid the management of antibiotic resistance by facilitating more targeted antibiotic use. However, many improvements are still needed in practice. The proposed interviews will inform the shaping of policy options to better support diagnostic innovation.

**Your participation**

You have been invited to participate in a research interview (of around an hour and a half). The interview will use the multicriteria mapping (MCM) method, an interactive method which allows you to explore alternative policy options for supporting the development and use of diagnostics to manage AMR, **using any criteria you choose** to appraise them in a systematic way. More information about the MCM method can be found on page 2. Please take the time to familiarise yourself with the policy options you will be considering during your interview (these can be found on pp. 3-4). Please also **consider in advance of the interview which criteria are important to you for judging these options** (p.5).

Following the interview, a record of your views and any accompanying notes will be stored on the computer of your interviewer. Quotes gathered throughout the interview will remain anonymous, unless you give explicit permission to use these. You will be notified if any of your anonymised quotes are to be used in publications stemming from this research. Interview recordings and transcripts will be destroyed after any minimum period of storage required by the project funder.

**This document**

This document provides you with an introduction to MCM, a list of policy options which you will be invited to consider during your interview, and details to help you to formulate your own criteria for judging the policy options to be discussed at the interview.

**Introduction to Multicriteria Mapping**

**What is MCM?**

Multicriteria mapping (MCM) is an interactive appraisal method for exploring contrasting perspectives on complex policy issues. The tool aims to help 'open up' technical assessment by systematically 'mapping' the views on alternative policy options and associated issues and uncertainties.

MCM enables participants to stay ‘in the driving seat’ in expressing their views while providing a qualitative and a quantitative perspective on a range of policy options. In doing so, this also allows rigorous comparisons to be drawn across different perspectives.

**The MCM interview process**

An MCM interview has four main stages (shown in light grey in the figure – to the right), although it is possible to go back and review, add or edit at any stage and there will also be time for consideration and reflection at the end.

The stages are:

- **Choose options** to be appraised
- **Define criteria** to be used for appraising options
- **Assess scores** of each option against each criteria
- **Assign weights** to each criteria

During an MCM interview, you will be given a list of predefined ‘core options’ (pp. 3-4), which represent a range of possible policies or other courses of action for achieving a particular aim.

**You can add to these and redefine them if you wish to, to create additional options**. Next, you use your own sets of appraisal criteria to evaluate the policy options and then assign optimistic and pessimistic scores under each criterion for each option (using any scale you wish to employ). At every step, care will be taken to record your reasoning, capturing important uncertainties and ambiguities that explain the performance you expect of policies under a range of circumstances. You will be asked to justify your scoring in relation to your own understanding of salient evidence. When all options have been scored under all criteria, you can then assign simple numerical weights to criteria to express their relative importance.

Aided by a visual representation of the overall option rankings based on the scores and weights, you will be invited to interact with this picture to help you settle on a final set of weightings and (if necessary) also revisit the scoring process until you are satisfied that the final picture meaningfully reflects your perspective on the performance of the various options that you have considered.

After a number of perspectives have been collected in this way, the research team will analyse qualitative and quantitative results to give a rich picture of different priorities, contexts, uncertainties, ambiguities and conditions under which contrasting policy options perform better or worse.

**Policy Options**

This section lists 6 ‘core options’ which all interviewees will be invited to appraise. Since this study is being replicated in different countries, you are invited to only consider your country’s context when discussing the options below. The following policy options were developed using an extensive literature review, combined with expert advice. You should familiarise yourself with the policy options before the interview. **The list of core options is not exhaustive and interviewees are welcome to add new options as they see fit during the interview.**

- **Enhance revenues**

**Key Features:** Government encourages firms to participate in the diagnostics market by increasing financial rewards.

**Description:** This option assumes that the market for the diagnostics needed to manage AMR is not sufficiently rewarding for firms, and that they do not engage in this market as much as would be beneficial. Government can provide additional financial incentives to encourage firms to invest more in developing new diagnostics for AMR by making the market more profitable. There are many mechanisms through which this could be done (e.g. prize funds, advanced market commitments, and new enhancements to intellectual property rights that provide rewarding periods of monopoly), but the common factor in these is that they increase public spending on diagnostics in order to more highly reward private provision of testing technology.

- **Fund R&D**

**Key Features:** Government encourages diagnostic innovation by providing researchers with more funding for R&D.

**Description:** This option assumes that there is a need for more knowledge or skills to develop new diagnostics to manage AMR. Governments or other organisations (e.g. charities) can provide grant funding for both public and private researchers to support the generation of new knowledge and skills. These funds are separate and additional to healthcare spending. Funding for research and development can take different forms. It is available to public and private sector researchers, as well as collaborations between public and private.

- **Making Pathways**

**Key Features:** Government coordinates stakeholders to provide help for firms seeking to bring new tests to market.

**Description:** This option assumes that it is often difficult or risky to commercialise diagnostic tests because healthcare systems are complex and firms cannot predict what these systems will need, or what standards of evidence are required for a diagnostic to be commercially successful. Regulators, payors/insurers, and healthcare systems can provide guidance and support to give test developers a clearer view of what is needed, and to help them to access the advice and resources they need to develop products, get products licensed for use, and obtain purchasing approvals.

- **Government provides**

**Key Features:** Government leads R&D and clinical testing to ensure optimal test use in their healthcare system

**Description:** This option assumes that profit-making firms will not produce the right tests at the right price or make these widely available enough. Government can directly facilitate the provision of all required resources as needed, for example through publicly funded laboratories, so that diagnostic services are made available and more affordable at the point of use than would be the case when relying on commercial providers.

- **Incentivise use**

**Key Features:** Healthcare providers create incentives and remove disincentives to encourage better use of tests.

**Description:** This option assumes users can make better use of available tests than is currently the case. Healthcare providers can provide incentives and remove disincentives to encourage test users to make more use of diagnostic tests to guide their clinical decision making around antibiotic use. Incentives include rewards or mandates that encourage test use. Removing disincentives could involve changes that ensure care decisions are not based on factors such as antibiotic drugs costing less than diagnostic tests.

- **Protected markets**

**Key Features:** New tests are developed based on market demand and established international IP protection regimes.

**Description:** The following is assumed to be already possible with established laws. The option assumes that the testing technology produced by firms is proprietary (based on current intellectual property protection), and that firms use this legal defence from competition as an opportunity to invest in novel diagnostic applications. Firms focus on building and maintaining their customer base and develop technologies that they hope will achieve this aim. The availability of a protected market leads to firms developing the diagnostics needed to manage AMR and customers’ willingness to pay for these tests rewards firms for providing them.

**Table of options organised by key features:**

| **Options** | **Stage of innovation process** | **Mechanism** | **Role of government / private industry** | **Actors of focus** |
| --- | --- | --- | --- | --- |
| - **Enhance revenues** | Downstream | Pulls suppliers towards the market with incentives | Government and private industry reliant | Diagnostics firms |
| - **Fund R&D** | Upstream | Pushes new technology towards the market | Government reliant | Public and private researchers, diagnostics firms |
| - **Make pathways** | Upstream | Signals to suppliers the needs of the market | Government and private industry reliant | Diagnostics firms |
| - **Government provision** | Upstream / Downstream | State provision of the required goods and services | Government reliant | Healthcare systems |
| - **Incentivise use** | Downstream | Encourages demand to grow by incentivising use | Healthcare system reliant | Clinical users |
| - **Protected markets** | Downstream | Pulls suppliers towards the market with incentives | Private industry reliant | Diagnostics firms |

**Choosing your appraisal criteria**

Criteria are of central importance for appraising the policy options during the MCM interview. They are used to determine which policy options are likely to perform poorly or well, according to your judgement. You are free to develop your own criteria for the appraisal of the policy options and are strongly encouraged to invest some time before the interview thinking about which criteria to use as this ensures a more satisfying interview experience.

Typically, participants will be asked to develop 3-5 criteria only. This helps avoid interview fatigue as each option needs to be appraised under each criterion. A prompt sheet will be made available to all interviewees in case assistance is needed with criteria, but you are entirely free to develop your own criteria without prompting. Each criterion should be as distinct as possible from other criteria.

All criteria should be relevant to your own view about what counts as ‘good’ or ‘poor’ performance of options. Every criterion will be applicable to all options. It is possible to express indifference with neutral scores and uncertainty as wide scoring ranges.

**Illustration of MCM criteria – the commute to work**

In order to assist you in thinking about suitable criteria for the MCM interview, the following illustration is provided. We use criteria that are unlikely to be relevant to the interview on AMR deliberately to minimise bias.

Consider how you commute to work. For example, imagine you can choose to ride a bicycle or drive a car or catch a bus to work. These would be ‘options’ in MCM.

Now consider some criteria that can be used to evaluate the options. Let’s assume you most value:

(i) the opportunity for physical exercise

(ii) chance to reduce local pollution

(iii) minimising travel time

The bicycle might be favourably appraised against the first two criteria but not the last, while the opposite would be true for the car. The bus may perform better overall, assuming you walk to the bus stop, even though it may not perform best by some criteria (MCM captures such qualitative information). Your ultimate choice may also depend on which of the criteria you value above all others – another choice that MCM invites you to consider.

**Annex C – Additional options and criteria**

This Annex lists the criteria and additional options specified by the interviewees in this study.

**Criteria**

Interviewees were free to define their own criteria by which to judge the policy options. The 47 interviewees defined 118 criteria. They are grouped thematically and listed in descending order of the number of people who defined criteria in each group. Where multiple people defined criteria in a group, multiple definitions are given below (although exact or very similar descriptions are not repeated).

| **Criteria group name** | **No.** | **Criteria group definitions – as provided by interviewees** |
| --- | --- | --- |
| Collaboration | 9 | Government and industry collaboration / Cooperation – including between firms for pooling resources / Collaboration, not only between the same speciality but between different specialists, e.g. medical doctors and technicians / Collaboration between industry and academia. For the development of new tests, there are different phases, idea, technology, phase one and two, then phase three clinical application, whether it gets back results expected. Indispensable for this is collaboration. / Alignment and collaboration between multinational, national, regional companies, and other stakeholders. They have different barriers and opportunities. This is about collaboration and alignment between the different stakeholders. / Public and private collaboration / Engaging different stakeholders, everyone integrated in the development of ideas, including appropriate use of resources. |
| Cost effectiveness | 9 | Cost effectiveness / Cost effective use of tax resources / Profitability for the company - companies need good profits to invest / Is it cost effective for the healthcare system? Does it help reduce costs of treatment? Is it provably worth investing in the sector to reduce costs elsewhere? / In the health system overall, it should be more cost effective / Health economics and mechanisms. |
| Costs | 9 | Costs / Cost of a diagnostic test / Cost to the healthcare system / Cost to health budget / Cost of implementation of the policy option / Cost to the GP / Cost of implementation / Cost of the entire process. |
| Control / Decrease AMR | 8 | Ability to decrease AMR / Will it be a game changer? / Impact on the problem / Protect public health from AMR / Stabilising or reversing increasing resistance / Will it change the situation? |
| Promoting health benefit | 8 | Promoting health benefit / Improve patient outcome / Clinical outcomes for patients, reducing morbidity and mortality / Improving patient management, outcomes such as length of stay, mortality, morbidity, quality of life / Does it improve medical care of patients? For example, for hospital infections, is it helpful in screening? Reducing time spent in hospital? Avoiding septic death? / Time in hospital, better management, including treatment. |
| Speed | 7 | Speed of innovation / Timeliness of results / Time to have permissions, time for approval, time to purchase tests / How long does it take before you have an effect on what you want to achieve, the end goal: to only apply antibiotics in the most rational way, to apply them only when needed / Depending on the option, these things take time, particularly at the EU level. Speed of both implementing policy right through to patients being tested. / Speediness of the development of the tests / Speed of the process. |
| Appropriate use of antibiotics | 5 | Can this policy option help preserve the effectiveness of antibiotics? In a technical sense. This is measurable as soon as a diagnostic is on the market. Does it really help for the problems we're facing? To manage the consumption and use of antibiotics / Appropriate use of antibiotics. This is particularly important in Greece. Just to give the antibiotics when it is really necessary. / We want to treat our patients with restricted/prudent use / Change patient pathway so as not to give out antibiotics if not necessary. |
| Change culture / increase awareness | 5 | Change culture and habits / Currently we think that AMR is a small problem. There is a law for all this but we don't follow the law. Infection control, AMR, to fight them in the hospital and in the community. We have all the tools to face the problem, but we don't use them. The law from 2015 EU legislation. / Applicability, you have to persuade people to do it / Awareness of healthcare professionals. Even if you have a new test on the market and a strategy, this should not come out of the blue. There are also political options to increase awareness. The whole topic benefited from the German Chancellor putting it on the agenda in an EU/G7 meeting. Such action would be helpful not only as a one-day topic but as a permanent topic so that the everyday physician is aware that we are running into problems with giving antibiotics to everyone. / We need a strong message, communication. The population should understand that government is trying to invest in something really important for the whole population. People need to be aware of the problem. |
| Cost benefit | 5 | It's not the cheapest, it’s about total cost-benefit, both funding and savings, return on investment, total cost to healthcare service. / Whether or not it leads to a direct increase to health budget compared to risks or benefits. / The relationship between the cost and the health-based benefits - quality of life. Impact on improved outcomes, not just a policy goal that spent a lot of money on improved accuracy of a test - but does that improve outcomes as well? / Maximising cost but controlling for health. |
| Feasibility | 5 | Is it possible? / Legal feasibility - there are some options that are difficult to put into place because it interferes with legal circumstances either nationally or at the EU level. This touches issues such as market access, copyright, granting extra money to a certain field, intellectual property rights. This is not fully possible for all options. / Organisation – is it possible to organise? This is about structures, e.g. for funding tenders, calls, the way the funding system is structured |
| Easy to use | 4 | Easy to use not only for clinicians, but also other healthcare professionals. The diagnostic has to be easy to use and accurate, and also easy to use for a wide range of practitioners (including nurses - these people identify the problem). / Practicality - simplicity - effective for everyone to use / Ambulatory care needs to be tested as soon as possible as there is saturation of the use of emergency services. This needs easy-to-use diagnostics. / Ease of access to clinical users widely and promptly. |
| Implementation | 4 | Implementation / How implementable are the policies? What is the process by which this becomes an active policy option? Implementability, barriers to implementation / Practical for GP, practical implications for GP, touchable and tangible outcomes. |
| Acceptability | 3 | General acceptability for stakeholders, acceptance or support from the different stakeholders. / Acceptability of the diagnostics for health care professionals / Will healthcare workers comply with the proposed option? |
| Effectiveness of diagnostics | 3 | Useful diagnostics, e.g. early diagnostics for the type of resistances, effectiveness. / Development, antibiotic accuracy, the diagnostic has to be right, faster. New diagnostics are needed, fast, to improve testing, and also the type of diagnostics, with more information. / Patient access to useful diagnostic tests. |
| Innovation | 3 | New innovative tests / New technology. |
| Necessity | 3 | Is it necessary? Is there a problem? / Is it needed in the community? / Is it needed in the hospital? |
| Does it fit | 2 | Does this option fit with the Dutch system? / Does it fit within the German health insurance and funding system? |
| Improvement of evidence | 2 | It is important that improvement in technology and awareness paralleled by improvement in the evidence base. Manufacturers are not thinking beyond CE certification. Once they have CE certification, they can enter the market, job done, but actually this will require real change in data practice, CE is not sufficient. Some of the options would lead to interesting products and market access but we have forgotten to plan ahead the evidence base. I am keen on having a robust evidence for diagnostic tests. / Prior evidence of effectiveness in a relevant field. |
| Market sustainability | 2 | Provide enough incentive, dependability and power/strength for manufacturers involved, not just one off, long term route to sustainable market / Specificity, particularly targeting market failure. |
| Quality | 2 | Quality / Prescription quality. |
| Standardisation | 2 | Standardisation of the procedures for the exams. There are many standards in measuring. In the literature there may be one measurement and in Greece a different measurement and this is confusing in primary care. Standardisation to have one measure. Standardisation of the tests and the exams and the laboratory values. / Homogeneity of diagnostics to use across all regions in Spain. |
| Complexity | 1 | The number of players involved, the level of action that needs to be taken to achieve the policy option. |
| Effectiveness | 1 | Effectiveness of the policy in doing the job without interference, without worry about the cost. Public health insurance, this regulates everything. In doing my job, effectiveness is because I wish to do my job without interference from health insurance. The system is over-regulated, and effectiveness has to do with over-regulation. Over-regulation has an impact on daily practice. |
| Effects | 1 | Cost for society or for patients or for healthcare providers. What are the effects of the test? Does it support treatment decisions or make people more dependent on healthcare system? How much would it lead to overuse of tests? |
| Engagement | 1 | Engagement from the government to sustain all we need for primary health. Engagement from colleagues to sustain the programme of the primary health so we can do our job. |
| Focus | 1 | Focus in the development of tests, e.g. so if you fund many firms, you may then end up with too many tests. |
| Government function | 1 | Government should support and promote but that's it. It doesn’t work to give a function to government which is not a government function. Government should support and promote the principle but that's it. |
| Identify specific antimicrobial | 1 | Identify the specific antimicrobial mediated - to reduce bad use and misuse of some antimicrobials, we have to identify the problem. |
| Limiting uncertainty | 1 | Limiting uncertainty - overuse of antibiotics is often due to uncertainty. |
| Policy led by microbiologists and clinicians | 1 | Policy should be led by microbiologists and clinicians - those sources. For example, there has been a 3-year fight about rapid tests. There have been clinical trials and tests have been developed but hospital management demand that we use them based on costs. Clinician-led, cost after outbreak - they should demand increased quality and be critical. |
| Regulatory and structures | 1 | Regulatory and structures |
| Removing barriers | 1 | Removing barriers |
| Risk | 1 | Risk to the company |
| Speed of diagnostics | 1 | Speed is important for clinicians, so you can change the antibiotic. This helps more with stewardship for use in hospitals. |
| Synergy capacity | 1 | If any of these stands on its own, that's not a good idea. |
| Tools - adapt response | 1 | Adapt tools to the magnitude of the problem. |
| Translation | 1 | Capacity to translate. You might create a pathway, but the chance of translating may be poor. Directness of incentive to increasing diagnostic use, e.g. it's very easy to create an R&D scheme but you may not see outcomes. |
| Understanding AMR | 1 | Understanding AMR better and its dimensions. Which dimensions are important? System thinking. |
| Usefulness | 1 | Usefulness for IVD companies. |

**Additional options**

Interviewees were also free to add their own additional options to appraise alongside the 6 core options. 47 interviewees defined 21 additional options. The additional options span a wide array of themes and so they have not been grouped thematically but listed alphabetically instead. The option definitions have been copied from the interviews.

| **Additional options** | **Additional option definition** |
| --- | --- |
| 24/7 labs | In Germany and many EU countries, microbiology labs work 10 hours a day and also on weekends and holidays but even shorter so there is a long delay from samples being taken 24/7 in hospitals until it reaches the microbiology lab and further processing can start. The average time for blood culture to be entered into the instrument is 13 hours, during the day 1-2 hours but there is 14 hours night silence and 18-16 hours weekend silence, so an average of a 12 hour delay. This is true for other specimens too. Using molecular tests that increase turnaround times is secondary to having a 24/7 lab where I could improve turnaround by 12 hours. Using the infrastructure we have already but we need more technical and academic personnel. If you have a 24/7 lab then molecular tests are much more useful than they are now. You can start the test soon after it's taken. The benefit of rapid tests cannot be exploited if you do not have 24/7 lab. |
| Aiming for a wider market | Companies also need to develop diagnostics for new markets. So the involvement of the new market for the development is needed, to aim for a wider market. |
| AMR stewardship | AMR stewardship with multidisciplinary personnel, intensive care units, microbiologists, pharmacists, and clinicians. In this option, the people can review weekly or daily the use of antibiotics, and develop guidelines of use for regional guidelines. It involves diagnostics microbiologists. They need to take the antibiogram, and they have to use diagnostics to understand what resistance is developing and what resistance has developed. If people have fast diagnostics, we can help infectious disease. We need to change the broad spectrum. Bring more quick information, no need to wait three days, 24h hour diagnostic is important. To impact the use as quickly as possible the right antibiotic. |
| Empowerment of patients | Educational programs for patients, e.g. the HIV test is available in pharmacy. It’s also about the availability of the test, patient use of the test, and benefit of test (education). |
| Enhance regulation | Enhance regulations for diagnostics. There should be clear and transparent regulations regarding entering diagnostics on the market, e.g. approval. Like the commission, which assesses new products and decides if they are allowed to enter the market. |
| EU joint clinical assessment | The EU joint clinical assessment is a new proposal coming from EU in terms of HTA pathways and in particular related to the joint clinical assessment. This could represent a very interesting pathway because as you know joint clinical assessment is strictly related only to the clinical characteristics of the products and making comparison with products which already exist on the market. So this could help the acceleration of market access because one of the most important hurdles, especially in Italy, depending on regions, because regions very often say 'ok, but this could a very interesting product but we would like to better understand which is the real clinical advantage, which are the most important adverse reactions', and so on and so on. When you have a joint clinical assessment result, it's more difficult for regions to continue to follow this approach. |
| Evaluation and translation | Diagnostics will be evaluated via large EU networks, shared patent with EC. Diagnostics companies are conducting their research in very well established clinical networks, de-risking. Then in terms of implementation, these are big pan-EU networks. Therefore the chance of implementation is enhanced. The downside is that you need to share your IP and profits. It's like pathways, not regulatory pathways, it's about clinical trials and implementation. |
| High quality tests | GPs need to know that the test is of high quality and very well tested. This needs collaboration with the government lab to certify the quality of the test. A certification to give a test a label. Collaboration between public lab and company. |
| Incentivise use – payers | Healthcare providers create incentives and remove disincentives to encourage better use of tests. The same as incentivise use but for the CCG perspective. |
| Independence of suppliers | Evidence base and independence of health suppliers. |
| Indirect initiatives | Indirect initiatives to optimise infection management. Like CQINs. |
| Information to GP | This involves both sharing information to GPs, sharing between GPs, and also getting epidemiology information about resistance, and epidemics so that GP can react more quickly. |
| Management of HR | Management of human resources, changing the culture, working time of microbiologists, continuity of resources, microbiologists do not work together. There is a great opportunity for technology. Also, time issues. |
| Market development | Market development - there are lots of small firms, very few large firms and it's hard to bridge that because large firms have a worldwide sales force. This would help bridge the gap. |
| New patient pathways | For example, MRSA diagnostics used to take 2-3 days, there was exponential growth across the UK, then we changed the pathway, Netherlands approach, guilty until proven innocent, stops the spread. |
| Patient involvement | Patient influence in the development of diagnostics, awareness, and what the patients think about AMR. |
| Raise awareness of the problem | The need for microbiologists, clinicians and the press to make pressure at the local level. |
| Raising awareness | Raising awareness, and providing information and education for the general public. |
| Regulation of use of antibiotics | In Germany if we want to prescribe opiates, we have to do this on a specific prescription form and this is evaluated. As a doctor I have to get these special forms and this is a good measure to minimise abuse. For certain antibiotics we need to regulate use in this way. It makes real time data available on specific antibiotic substances. If we try to implement in these countries, which are very different, if we have feedback on just fluoroquinolones or new antibiotics, then we have to be reluctant to use them but of course this is against the interest of the company developing them. Companies can market new drugs for general use when that is not appropriate. |
| Technology transfer | Providing support for researchers to move their scientific discoveries or inventions towards further development. |
| Training | Training doctors, patients too, multi-plain activity, and policymakers. |

**Annex D: Diagnostics for AMR – A crowd sourced checklist for effective policy implementation**

The following checklist has been developed as a point-by-point response to suggestions and concerns raised by stakeholders in relation to the design and implementation of policy instruments to support diagnostic innovation for the management of AMR. Contributing groups included primary and secondary care clinicians, clinical laboratory scientists, pharmacists, diagnostics industry executives, HTA experts and policymakers in six European Countries (Germany, Greece, Italy, the Netherlands Spain, and the UK). The points raised indicate concerns from different perspectives and in settings where national AMR plans are at different levels of implementation, hence these suggestions may not be universally relevant. For this reason, this checklist should be understood as a series of prompts, which should be considered locally, rather than a list of recommendations.

**Policy interventions to ‘incentivise the use’ of diagnostic tests**

(whereby healthcare providers create incentives and remove disincentives to encourage better use of tests)

- Will reimbursement of diagnostic tests leave prescribers with higher costs than using antibiotics alone?
- Will guidelines change/be provided on treating diseases, using medicines and using tests?
- Will you provide guidelines, education and training along with requests for greater test use?
- Will your implementation strategy include a communication strategy for dissemination of guidelines?
- Is a change management process needed to stimulate change in culture and behaviour around diagnostic test use?
- Isn’t it better to avoid punitive actions?
- Will the financial incentives be sufficiently large to work, and can they be funded over the long term in a sustainable way?
- Are stakeholders coordinating at the national and local levels?
- Are stakeholders aware of the importance of coordinating here?
- Are stakeholders involved in the design of the incentives policy?
- Are there barriers preventing users and test developers from communicating?
- Are users involved in developing tenders for public procurement?
- Can the use of existing tests be incentivised for rapid effect?
- Will the incentive scheme be easy to use? Or do users think it is too bureaucratic?
- Will tests be readily available at all times?
- How stable is the supply of tests?
- Will costs be transparent to stakeholders?
- Will an appropriate range of tests be available?
- Will tests fit easily within existing clinical routines?
- Will prevalence data on AMR be shared to aid decision making?
- Will incentives work at the level of health professionals, healthcare organisations, health insurers and diagnostics firms?
- Is the implementation strategy detailed enough?
- Do you have a sufficient infrastructure to support testing, for example including transport and IT?
- Should use of new antibiotics have mandatory testing?
- Is there sufficient harmonisation of testing guidelines across organisations and regions?
- Would it be better if healthcare providers had control over procurement of tests?
- Will this incentive scheme to be linked to other relevant pathways?
- Is there evidence of clinical utility for the specified diagnostics?
- Has relevant evidence underpinning this incentive scheme been widely shared with relevant clinicians?
- Are there clear benefits to patient from using tests?
- Do the available tests aid clinical decision making?
- Should healthcare workers change their routines to accommodate the use of testing?
- How can those reluctant to use tests be persuaded?
- Are the tests viewed as being of good quality?
- Do users think these are the right tests for the context?
- Acknowledge where a suitable test is not available
- Can healthcare providers plan ahead adequately with the proposed incentive scheme?
- Identify and acknowledge uncertainties
- Do the proposed incentives favour the needs of the community over profit for companies?
- How can the incentives be associated with the building of trust?
- Will incentive schemes address areas of greatest need?
- How will the incentive schemes affect smaller organisations? (hospitals and companies)

**Policy interventions to fund R&D into diagnostic tests**

(whereby government encourages diagnostic innovation by providing researchers with more funding for R&D)

- Will R&D funding encourage collaboration between public and private actors, with appropriate oversight of pubic money?
- Will R&D funding include clinical utility/validation and cost effectiveness research to provide sufficient evidence to encourage test use?
- Is the budget for R&D funding sufficiently large to make a difference?
- Is funding R&D cost effective? How can this be measured?
- Since funding R&D takes a long time, can R&D be funded continuously over the long term in a sustainable way?
- Will the process for funding R&D be easy to use? Or do users think it is too bureaucratic?
- Should diagnostic test R&D include a range of types of diagnostic tests for different end users?
- Will patients be involved in diagnostic test R&D?
- Will the R&D funding scheme provide clarity to both public and private actors about what will be funded so that they can plan ahead?
- Will funding be provided to poorer regions to allow them to harmonise with other regions?
- Is there a robust system for evaluating research?
- Will you provide carefully designed R&D questions to encourage rapid results?
- Is R&D funding linked to implementation and uptake strategies?
- Are there control mechanisms built into the funding scheme to ensure funding is spent on R&D, to ensure results are produced, and to combat corruption?
- Will GPs and other healthcare professionals be involved in R&D projects?
- Will you look ahead to which resistances are likely to cause problems in the future as well as funding research on current problems?
- Should the R&D funding not be directly only to companies?
- Are there ways to increase the ranking of AMR-related research to encourage researchers to join/stay in the field instead of addressing other priorities?
- Are the R&D evaluators experts in the field?
- Are there ways to encourage patient involvement to overcome perceived inconvenience?
- Will you provide longer term contracts for researchers to ensure continuity?
- Have you considered the important role played by microbiologists?
- Are there ways of ensuring that the test systems which are developed are easy to use?
- How should the R&D funding be distributed? How can you ‘pick winners’?

**Policy interventions to ‘make pathways’ to support the development diagnostic tests**

(Whereby government coordinates stakeholders to provide help for firms seeking to bring new tests to market)

- Is there good coordination between different stakeholders?
- Will the initiative to make pathways create consensus between different stakeholders?
- How will making pathways help stakeholders to navigate the complexities of the system?
- Will you provide clear, evidence-based guidelines as part of this scheme?
- How can you ensure guidelines are implemented?
- Will you provide clear definitions of the standards of evidence required to prove clinical utility of tests and encourage test use?
- Are there ways to measure the cost effectiveness and impact of making pathways?
- Will you build in appropriate timescales to ensure that making pathways contributes to speeding up the path from development to market?
- Should making pathways include an educational element to raise awareness about AMR?
- Will the initiative to make pathways be integrated with funding R&D because we need discoveries and implementation?
- Can coordination between stakeholders contribute to making clear criteria for R&D?
- Will you provide long term guidance on the conditions for reimbursing diagnostics so that industry can plan ahead?
- Will diagnostic firms be better joined with end users?
- Will (smaller) firms be helped to understand how to enter the market and what evidence to provide at what stage, e.g. randomised controlled trials?
- Will long term political support be maintained to ensure long term sustainability?
- Will appropriate information be provided to different stakeholders?
- Should companies be encouraged to provide not only testing but information and informatics, provided in a smart manner?
- How can the needs of patients and test users be better understood and translated?
- How can testing be adapted towards the needs of patients and test users?
- How can the process be simplified?
- Will making pathways encourage harmonisation between different regions so that efforts are not duplicated?
- Can making pathways ensure quality of diagnostic tests?
- Will pathways be easy to follow to reduce uncertainty, ensure cost effectiveness and facilitate the use of tests?
- Will you provide assistance with regulatory and legal issues, and paperwork?
- Can pathways be designed within a One Health concept?
- Will accreditation of tests be developed to encourage the uptake of novel tests, e.g. an ISO norm?
- Should data sharing be encouraged between stakeholders?
- Will the model span disciplines and professionals, e.g. to link doctors with veterinarians and pharmacists?
- Will you consider the flow of making pathways?
- Will you identify particular barriers / challenges and address them?
- Will you implement this option in combination with other approaches?
- How can you ensure different actors engage with efforts to make pathways so that this initiative has an impact?
- Should regulatory pathways be improved?
- Should making pathways include guidance and tools to aid implementation and marketing, as well as improving regulatory pathways to get a CE mark or FDA clearance?
- Acknowledge that what is feasible depends on the macro-economic situation.
- Will support be provide for test developers to provide appropriate evidence for the context, e.g. in the German context new diagnostics must be shown to make a difference in the treatment chain, which is not easy to isolate?
- How can you ensure that industry, HTAs and clinicians understand the evidence that is needed?
- Will making pathways work within current systems and with current policies to ensure crossing or merging streams rather than poorly overlapping parallel streams?
- Will company-involvement be encouraged where it is needed?
- Acknowledge the uncertainties associated with making pathways as an indirect initiative.

**Policy interventions to ‘enhance the revenues’ associated with diagnostic tests**

(whereby the government encourages firms to participate in the diagnostics market by increasing financial rewards)

- How can an initiative to enhance revenues ensure communication and collaboration between public and private actors?
- Will cost and cost effectiveness of enhancing revenues be evaluated as part of this initiative?
- How can you address concerns from some actors that government should not provide additional funding to companies?
- Will you build in processes to ensure societal benefit for all and not just firms maximising profits?
- Will enhancing revenues address uncertainties about patient needs and resistance?
- How can enhancing revenues ensure quality of evidence, clinical utility and quality of product?
- Will you provide guidelines on how to value diagnostic tests?
- Will you define a scheme to enhance revenues for tests which are really needed?
- Will the scheme operate in a well-regulated environment, independent from government and other stakeholders?
- Will companies' role in healthcare be not only financial?
- Will this have a positive effect on the economy?
- Should tests be distributed freely, accompanied by an educational programme and should physicians be convinced through high quality evidence?
- Should enhancing revenues be more about value than cost?
- Should the tests developed as part of this scheme be simple to use and easy to interpret?
- How can you ensure that a scheme to enhance revenues is implementable?
- How can implementability be evaluated and rewarded?
- Should there be competitive tendering?
- Should timeliness should be considered as part of the scheme?
- Where healthcare is devolved to regions, how can you ensure communication and collaboration between regions?
- How can you ensure that tests are of high quality to be approved?
- Will there be an accompanying awareness programme?
- Will stakeholders receive information about the AMR problem and the benefits of optimising treatment by using tests to stakeholders?
- How can you ensure that enhancing revenues does not create an imbalance in the system?
- Will efforts be made to understand industry, the rules they play by and the negotiations that would be needed?
- How can you combat the lack of experience in implementing this type of policy?
- Will a sufficiently high level of scrutiny be given to enhancing revenues?
- How can you address concerns from healthcare providers about giving money to industry?
- Have you considered anti-competition law?
- How can you ensure this does not antagonise other initiatives?
- Have you considered the appropriate size and mechanism of enhancing revenues for SMEs as well as for larger firms?

**Policy interventions for government provision of diagnostic tests**

(whereby government leads R&D and clinical testing to ensure optimal test use in their healthcare system)

- Will government provision be cost effective?
- Should governments be in control of the whole process?
- Should government take the lead on pricing and reimbursement?
- Should government lead on improving access to laboratories and bacteria
- Should government lead on improving the quality of clinical testing and evidence?
- Should government lead on regulating the connection between industry and providers
- Should government lead on R&D?
- Should government lead on surveillance?
- Should government lead on setting incentives and a supportive framework?
- Should government regulate, manage and make policies?
- Should government lead on providing clear guidelines, education and training?
- Should guidelines include when, how and to which population?
- Would government provision be desirable if there is a national crisis, a change in political will, a market failure or for particular cases?
- Does government have the capacity and necessary capabilities?
- Is the necessary infrastructure in place?
- Is there appropriate collaboration between stakeholders?
- Would collaboration from the beginning of the process allow government to shape the system more?
- Will this initiative include independent advice from scientific and industry stakeholders?
- Will this initiative include research, training, reducing the costs and improving the availability of tests?
- Should government provision be focussed and encourage optimal test use and targeted therapies?
- Should government provision include a country-wide plan to reduce duplication between different regions and hospitals?
- How can government provision ensure standardisation?
- Will stakeholders get local data back to be able to analyse what happens to local resistance over time if rapid testing is introduced?
- How can you convince professionals that government involvement would work, e.g. success with HIV in Spain, rare genetic testing in the NHS?
- Can these examples be used as models to help design and implement this scheme?
- Will there be a national plan and/or a government decree?
- Will there be targets, incentives and synergies with other initiatives?
- Will timeliness be considered?
- How can you address concerns of stakeholders that this is not the role of government?
- Will you secure EU approval? In the EU context, government provision is difficult to put in place because it interferes with the market.
- Is there strong and sustainable government interest as well as public support?
- Can mechanisms be put in place to support this beyond the next change in government?
- Have you made provisions for change management?
- How can bureaucracy be minimised?
- How can you ensure that all relevant stakeholders are involved?
- Is there data to support this initiative?
- Have you considered the complexities and issues around implementation?

**Policy interventions for protecting markets for diagnostic tests**

(whereby new tests are developed based on market demand and established international IP protection regimes)

- Acknowledge that protecting markets is considered by some to be necessary but not sufficient on its own.
- How can you ensure that protecting markets does not mean too much money being paid to industry?
- Should protecting markets be done alongside other initiatives?
- How can you ensure that protecting markets works well in terms of encouraging diagnostic innovation to manage AMR?
- How can protecting markets address concerns about sustainability, improving evidence, patient access and preserving effectiveness?
- Can protecting markets increase the speed of diagnostic innovation?
- How can you incentivise game changers?
- How can you encourage standardisation?
- Should companies be educated about their responsibility, e.g. how many lives you’ve saved in the last 10 years?
- How can this include adequate clinical trials?
- Should there be additional criteria for diagnostic tests to meet and not only price, e.g. clinical utility, user needs?
- How can protecting markets address concerns that it is difficult to protect medical devices in the same way as drugs because the speed of innovation is fast and the type of IP is difficult to protect?
- Is protection worldwide since the diagnostic market is global?
- Is 20 years too long for diagnostics because the speed of innovation is faster?
- Will this stop firms from making good tests that are out of patent?
- Should there be an exclusive relationship between provider and market in some cases?
- What is the right balance between competition (which encourages diversity and innovation) and finding the best option (which encourages standardisation and cost effectiveness)?
- How does this affect smaller companies?
- How can awareness be increased?
- How can you address concerns about the acceptability of protecting the market?
- How can you ensure that tests are not too expensive?
- How can you ensure collaboration between different actors?
